# Dual Inhibitors of AChE and BACE-1 for Reducing Aβ in Alzheimer’s Disease: From In Silico to In Vivo

**DOI:** 10.3390/ijms232113098

**Published:** 2022-10-28

**Authors:** Noa Stern, Alexandra Gacs, Enikő Tátrai, Beáta Flachner, István Hajdú, Krisztina Dobi, István Bágyi, György Dormán, Zsolt Lőrincz, Sándor Cseh, Attila Kígyós, József Tóvári, Amiram Goldblum

**Affiliations:** 1 Molecular Modeling and Drug Design Lab, Institute for Drug Research, The Hebrew University of Jerusalem, Jerusalem 9112001, Israel; 2 Department of Experimental Pharmacology, National Institute of Oncology, H-1122 Budapest, Hungary; 3 KINETO Lab Ltd., H-1032 Budapest, Hungary; 4 TargetEx Ltd., H-2120 Dunakeszi, Hungary; 5 Institute of Enzymology, Research Centre for Natural Sciences, Hungarian Academy of Sciences, H-1117 Budapest, Hungary; 6 Department of Tumor Biology, National Korányi Institute of TB and Pulmonology, H-1121 Budapest, Hungary

**Keywords:** multi-targeting, acetylcholinesterase (AChE), β-secretase (BACE-1), dual inhibitors, in silico, enzyme inhibition, in vitro, in vivo, amyloid beta

## Abstract

Alzheimer’s disease (AD) is a complex and widespread condition, still not fully understood and with no cure yet. Amyloid beta (Aβ) peptide is suspected to be a major cause of AD, and therefore, simultaneously blocking its formation and aggregation by inhibition of the enzymes BACE-1 (β-secretase) and AChE (acetylcholinesterase) by a single inhibitor may be an effective therapeutic approach, as compared to blocking one of these targets or by combining two drugs, one for each of these targets. We used our ISE algorithm to model each of the AChE peripheral site inhibitors and BACE-1 inhibitors, on the basis of published data, and constructed classification models for each. Subsequently, we screened large molecular databases with both models. Top scored molecules were docked into AChE and BACE-1 crystal structures, and 36 Molecules with the best weighted scores (based on ISE indexes and docking results) were sent for inhibition studies on the two enzymes. Two of them inhibited both AChE (IC_50_ between 4–7 μM) and BACE-1 (IC_50_ between 50–65 μM). Two additional molecules inhibited only AChE, and another two molecules inhibited only BACE-1. Preliminary testing of inhibition by F681-0222 (molecule 2) on APPswe/PS1dE9 transgenic mice shows a reduction in brain tissue of soluble Aβ42.

## 1. Introduction

AD (Alzheimer’s disease) is a neurodegenerative disease that is responsible for ~60% of cases of dementia and is characterized by loss of memory and other cognitive disabilities [1,2,3,4]. It is estimated that more than 50 million people worldwide suffer from dementia and this number is expected to grow and involves very high costs of medical care (in the trillions of dollars) with yet no cure [5,6]. There are currently four approved drugs that help to relieve AD symptoms, including three AChE (acetylcholinesterase) inhibitors: donepezil, rivastigmine and galantamine, and one NMDA (N-methyl D-aspartate) antagonist: memantine. These drugs do not cure or inhibit the development of AD, and therefore, there is a great need for new drugs that prevent, delay the onset, slow the progress or treat the symptoms of AD [4,5].

The two main theories on the origin of AD are the “amyloid theory” and the “Tau theory”. In some cases, AD pathologies are the result of genetic factors, while others are: aging, head injuries, diabetes, smoking, obesity, etc. [7,8,9,10]. One of the main suspects for the onset and development of AD is Amyloid-beta (Aβ) peptides. Therefore, the reduction of its formation, aggregation and sedimentation are some of the main goals in the development of a cure for AD. Aβ peptides are formed by consecutive cleavage of APP (amyloid precursor protein) by two enzymes: β-secretase (or beta-site APP cleaving enzyme 1, BACE-1) first and, subsequently, by γ-secretase. In addition, it was suggested that another enzyme, AChE, is involved in Aβ aggregation [5,11,12]. Therefore, inhibition of one or more of these three enzyme actions may help to stop the process of Aβ formation/aggregation.

Inhibition of two or more biomolecular targets with one drug (a single molecule) is part of a more recent therapeutic approach known as “multi-target directed ligands” (MTDL approach), which gains much interest in multi-factorial diseases such as AD [13]. It is of interest also in the case of natural products [14]. The MTDL approach is suggested to have better efficacy and safety compared to the traditional “single target-single drug” approach, as well as to combination therapy (“drug cocktail”) [4,13,15,16,17]. However, designing or discovering MTDL molecules is quite challenging since they are meant to bind to two or more targets, which may require different ligand properties for binding at each binding site [13,18,19].

**Figure 1:**
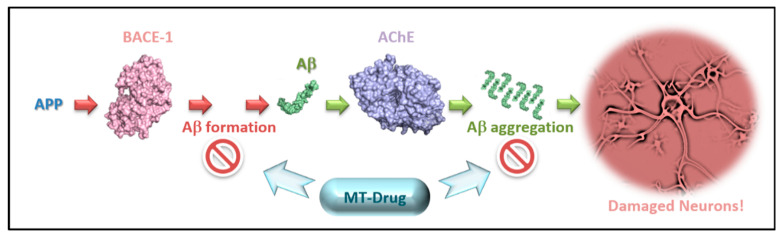
A MT (multi-targeted) drug could bind to both targets, BACE-1 (pink colored) and AChE (violet colored), so the two processes: Aβ peptide (green colored) formation from APP and Aβ aggregation. would be blocked. Additionally, by blocking AChE in the PAS, the level of non-hydrolyzed acetylcholine neurotransmitter would rise, thus, improving cholinergic activity simultaneously with the reduction in aggregation. The protein illustrations were made with Pymol software, Schrödinger and the PDB structure codes: 4M0F (AChE), 4DJW (BACE-1) and 1IYT (Aβ42).

Computational methods, such as docking, pharmacophore modeling and fragment approaches, as well as classification models and combined methods were already applied to MTDL discovery [13,20,21]. We chose to focus on two AD related targets, AChE and BACE-1, in order to discover novel dual inhibitors (Figure 1) for them. Inhibition of the catalytic site of AChE was used in the clinic to increase the amounts of its substrate, acetylcholine, thus, improving cholinergic transmission [22]. AChE is still the main clinical therapeutic target for treating symptoms of AD. More recently, it was suggested that the “peripheral anionic site” (PAS) of AChE is involved in the aggregation and formation of toxic Aβ oligomers [11,12]. PAS contains a large set of mostly aromatic residues, which present negative charges of π-electrons in their aromatic rings at the entry path to the AChE catalytic active site (CAS). Therefore, blocking the PAS could serve to reduce Aβ aggregation. In order to increase the chance to prevent harmful effects, we search for candidates to block the PAS (more details about PAS in Supplementary Word File, Figures S1 and S2) and simultaneously inhibit BACE-1 to prevent the initial cleavage of APP by that enzyme, which precedes the cleavage by γ-secretase to form the Aβ peptides (Aβ42, Aβ40) [23]. The 42-mer peptide, Aβ42, is the more toxic among these two. However, since BACE-1 has several substrates in addition to APP, which are not to be blocked [24], we suggest testing the effect of partial BACE-1 inhibition by weaker inhibitors, if obtained, which may be very effective despite their lower affinity to the target [23,25]. The need for partial inhibition was already evident with the failure of the highly effective γ-secretase inhibitor semagacestat, due to the total blocking of that enzyme, which is responsible for cleaving other substrates [26].

Most of the attempts to design dual inhibitors were focused on combining two scaffolds: one that binds AChE (e.g., tacrine, or N-benzylpiperidine moiety of donepezil) and another that binds BACE-1 (e.g., oxo-chromene) to form one molecule with dual activity [27,28]. Another study combined scaffolds of AChE and BACE-1 ligands by overlapping them to produce new dual inhibitors [29]. In some cases, the combination of scaffolds did not include a BACE-1 binding moiety, but the candidates were still tested for inhibition of that enzyme [30,31,32,33]. Contrary to much computational work for discovering AChE inhibitors, [34,35,36,37,38] there are few publications on computational methods for identifying dual inhibitors of AChE and BACE-1 that were also tested in vitro [39,40].

Here, we present a method for identifying dual inhibitors by screening large databases through ligand-based classification models, followed by docking the top candidates into crystal structures of both protein targets.

Our efforts led to the final purchase of 36 candidate inhibitors out of virtually screening ca. 3.2 million molecules from various databases. In vitro experiments identified two novel AChE and BACE-1 dual inhibitors, with one of the two also showing in vivo activity by inhibiting Aβ42 aggregation in a mouse model. Additionally, in vitro testing of the 36 predicted inhibitors found that some of them were active on one of the two targets and not on the other (AChE or BACE-1).

## 2. Methods

### 2.1. Preparation of the Classification Models and Screening

#### 2.1.1. Initial Preparation and Filtration of Datasets

Datasets containing BACE-1 and AChE inhibitors (for homo sapiens) were downloaded from the ChEMBL database [41]: BACE-1 data in November 2012 and AChE data in February 2014. Both underwent the rejection of duplicates, excluding molecules with too high activity values. Further processing was performed in order to reduce the bias of our modeling, which might be due to high similarity between the inhibitors. We identified similarity by calculating the Tanimoto index (TI) between all pairs of molecules. We examined the effect of molecular diversity by applying several cutoffs of TI values in the range of 0.7–1.0 and rejecting molecules that had TI above the cutoff values. Tanimoto indexes were calculated by OpenBabel using FP2 fingerprints. Models were built for each diversity value, and the models were evaluated by statistical criteria (see Table 1 and Table 2 in the results section).

The AChE dataset was filtered to include only non-covalent inhibitors, which are reported as binders to the enzyme’s PAS. This was performed by removing SMILES strings that included a phosphorous atom or carbamate functionality, as well as some esters, which are typical in covalent inhibitors. The identification of PAS binders was based on the literature referenced in ChEMBL and according to visual inspection of the molecules. (More details on dataset preparation and removal of non-PAS binders are presented in Supplementary Word file, Section 1.1).

#### 2.1.2. Preparation of the Training Sets

Two types of classification models were constructed: an “actives vs. inactives” (AvI) model and a “high vs. low” (HvL) model, which was constructed only from active molecules. Specifically, the AvI model consisted of active molecules (with an IC_50_ upper cutoff of 10,000 nM) heavily diluted by random picking of molecules from the ZINC database [42], while the HvL was a “fine tuning model” consisting of highly active molecules (IC_50_ < 100 nM) vs. molecules with low activity (IC_50_ > 1000 nM).

In order to build a classification model, each molecule in the datasets was presented by a class index: 1 or 0, for “active” and “inactive” (or “less active”), respectively. For AvI, the inactives were randomly picked from chemical catalogs (usually diluting the actives 100:1). For HvL, the model was constructed only with known actives—highly actives being one class and low actives as the other class. For AvI, we restricted the random picking of molecules to those that had some properties that were close to the average of each of four properties of the actives: H-bond donors and acceptors, molecular weight and calculated logP, according to the important principle of “applicability domain” [43]. Some 200 physico-chemical 2D properties were calculated by MOE (molecular operating environment, 2011 version) [44] for each molecule of the “actives” (from ChEMBL database) as well as for “inactives”, which were picked randomly from the ZINC database downloaded in 2011 [42]. Subsequently, properties were rejected based on either low variance or on high correlations between columns of these properties (details in Supplementary Word File, Section 1.2). KNIME software version 2.5.4 [45] was employed for these exclusions.

In HvL models, there was a relatively small difference in the number of molecules in each class. In AvI, the dilution of the actives with large numbers of inactives (“randoms” or “decoys”) was in order to “mimic” to some extent the process of “wet” high-throughput screening, which frequently finds a very small number of actives among a huge number of real tested compounds [46]. Here, the AvI models were built with ca. 35–240 times more inactives (randoms) than actives. It was expected that AvI may produce better models because there was a large difference between the actives and the randomly picked molecules from a large “chemical space”. HvL models were expected to be poorer than AvI models due to the greater similarity in HvL between molecules, which were all actives on a specific target.

In each of the models, we divided the learning set of the two classes (in AvI and in HvL) into five “folds”, which were picked randomly from each class so that each fold had exactly the same proportion of “actives” to “others” as the larger “learning set”. Every set of four out of the five folds was used, subsequently, to produce a model, and the remaining fold was used to test the model. This was repeated five times so that each of the folds served once as a “test set”. The main reason for that process was to reduce the chance of any bias by requiring similarity in the statistical parameters of all resulting models. (More details in Supplementary Word File, Sections 1.2 and 1.3 and Excel File, Tables S1E and S2E).

#### 2.1.3. Model Construction and Evaluation

Our in-house ISE (iterative stochastic elimination) algorithm was used to construct classification models made of multiple filters, each filter included five ranges of values of different properties. Each filter had a score reflecting its ability to identify correctly “actives” and “inactives” (or high- vs. low-activity molecules) on a specific target. That ability is expressed by the MCC value (Matthews’s correlation coefficient, Equation (1)) [47]. It was suggested to prefer using MCC to evaluate the performance of binary classification algorithms, in particular if unbalanced sets are to be predicted [48,49,50].

Models were evaluated by their MCC value and by their AUC (area under curve) as given for the combined five test sets. MCC scored the ability of the model (or a single filter) to correctly identify P (“Positives”, actives) and N (“Negatives”, inactives) or to incorrectly assign them (P_f_ and N_f_). More details about the ISE method, ISE output and model validation are in Supplementary Word File, Sections 1.4 and 1.5.

Equation (1):

(1) $$\mathrm{MCC}=\frac{{\mathrm{PN}-P}_{f}N_{f}}{\sqrt{{(N+N}_{f}{)(N+P}_{f}{)(P+N}_{f}{)(P+P}_{f})}}$$

P and N are the percentages (or fractions) of true positive and true negative predictions, whereas $P_{f}$ and $N_{f}$ are percentages of false positives and false negatives, respectively.

#### 2.1.4. Screening External Databases with ISE Models

The best AChE and BACE-1 models were used to screen and to score molecules from several databases: Enamine [51], ChemDiv [52], DrugBank (approved and experimental) [53], and three natural products’ databases, including: IBS [54], Princeton [55] and Analyticon [56]. Initially, 2D MOE properties were calculated for each molecule in these databases. The screening produced indexes (Equation (2)) for each molecule, one index for each selected model. The molecular databases were screened by five models, so each *molecule* has five indexes: two for BACE-1 models and three for AChE models. Specifically, the first filtration was through AChE model 1, and the molecules that received a positive index were then filtered through all the other models in parallel. Then, several selection conditions, based on these indexes, were used to pick the best molecules for subsequent docking. (For the conditions of selection and lists of selected molecules from the different databases, see Supplementary Excel File, Tables S3E–S9E and Supplementary Word File, Section 1.6).

Equation (2):

(2) $$\mathrm{Index}=\frac{\sum_{i=1}^{n}{\;\delta }_{i}\frac{P_{i}}{P_{\mathrm{if}}}{-\delta }_{\mathrm{if}}\frac{N_{i}}{N_{\mathrm{if}}}}{n}$$

where n is the number of filters. For each filter, if the molecule passed as active, δi = 1 and δif = 0, but in case the molecule did not pass and was found inactive by the filter, δi = 0 and δif = 1. P_i_ is the percentage of active molecules predicted correctly in filter i, Pif is the percentage of inactive molecules predicted incorrectly as actives in filter i, Nif is the percentage of inactive molecules predicted correctly in filter i, and Ni is the percentage of active molecules predicted incorrectly in filter i.

### 2.2. Docking Preparations and Screening by Docking

#### 2.2.1. General

The docking process included several pre-docking preparations: finding the best structure for docking from the existing crystal structures for each of the targets and then preparation of the structure (e.g., protonation states of relevant residues) followed by the docking procedure and the analysis of results. Our approach is different from simply using docking scores by the docking algorithms. We examined many PDB structures of BACE-1 and AChE with ligands and produced a table of all the residues that interact with all ligands, either by H-bonding or by Van-der-Walls (VdW) interactions. The residues that were found to interact with ligands in more crystal structures were, subsequently, used by us to validate the results of the docking. For more details on the docking preparations and procedure, see in Supplementary Word File, Sections 1.7–1.10.

#### 2.2.2. Selection of a Crystal Structure for Docking to BACE-1

Out of 75 human BACE-1 crystal structures examined for their interactions with ligands by the presentations at the PDBSUM site [57], 21 structures with resolution < 2 Å and with no mutations or missing segments were selected, downloaded from PDB [58] in 2013 and superimposed in MOE to find the two most different structures according to a pairwise RMSD matrix. Then, a test set of known BACE-1 inhibitors with high and low activity was docked to both structures (4DJW and 2G94) in order to find which one provides the largest number of true positives and smallest number of false positives. Ligand preparation, crystal structure preparation and docking were performed with OMEGA from OpenEye [59] to generate 200 conformations per molecule, using “MAKE RECEPTOR” for crystal structure editing and “OEDocking” for the actual docking [60] (Supplementary Word File, Section 1.7 and Table S1).

#### 2.2.3. Selection of a Crystal Structure for Docking to AChE

The AChE structure was chosen out of the few structures of human AChE that have resolution < 3 Å and a ligand bound in the PAS. The three structures’ codes with these requirements: 4M0F, 4M0E [61] and 4EY7 [62], had chain breaks between Pro258-Asn265 and Asp494-Pro498, which are both far from the binding site. Those missing coordinates were constructed by the MOE menu for structure preparation. Then, a test set of 10 AChE active molecules and 100 random molecules picked from ZINC was docked to these three structures and the one that had the best specificity (best identification of true negatives) was chosen for screening. (Supplementary Word File, Section 1.8 and Table S2).

#### 2.2.4. Crystal Structures Preparation for Docking

The structures were prepared by the MOE menu for structure preparation, removing duplicate chains, water and ligands. For BACE-1, three protonation states of the 4DJW structure were prepared: doubly deprotonated structure (i.e., deprotonation of Asp32 and Asp228), singly protonated Asp32 (ionic Asp 228) and another Asp32-protonated structure, by activating MOE’s “protonate 3D” protocol, which determines ionization and tautomerization of all other protein residues. These states of protonation were selected out of several options that exist in the literature [63,64,65] (Supplementary Word File, Section 1.9). For the AChE selected structure, 4M0F, several important residues were assigned neutrality: [66] Asp74, Glu202, Glu292, Arg296, Glu450 and His447, and Glu334 was negatively charged.

#### 2.2.5. Docking and Analysis of the Results

Prior to docking, duplicate molecules from the different databases were removed, in addition to molecules that were identified as mutagenic by MOE. The library of molecules to dock was prepared and minimized by MOE. Molecules with the highest ISE model indexes were docked by FlexX [67] to the chosen AChE and BACE-1 crystal structures, 4M0F and 4DJW, respectively. For each molecule, 10 poses were allowed. Default parameters were used according to the original FlexX paper [68].

The analysis of docking was based on counting the pre-determined main binding interactions: the Van der Waals contacts and hydrogen bonding, in order to decide which molecular poses could provide the required interactions that mimic those of ligand-bound crystal. Specifically, residues that were involved in hydrogen bonding to ligands in most of the examined BACE-1 crystal structures were: Asp32 (catalytic), Gly34, Thr72, Gln73, Asp228 (catalytic), Gly230, and Thr232. The residues that were found to be involved in VdW close contacts in most examined BACE-1 crystal structures were: Gly11, Leu30, Ser35, Tyr71, Phe108 and Thr231 (Figure S7 and Table S3 present alternative residues’ numbering).

The most important residues in AChE were picked by examination of 22 AChE structures including 2 human, 5 mouse and 15 Torpedo Californica or Tetronarce AChE PDB structures with ligands. The residues that created close contacts in most of AChE crystal structures were (numbering according to the human structure): Tyr72, Trp86, Gly121, Tyr124, Glu202, Trp286, Tyr337, Tyr341 and His447 (Figure S8). In AChE, these VdW interactions were much more noticeable than hydrogen bonding, so the latter interactions were not included in the criteria for analyzing the docking results.

Scripts written in-house were used to identify and to count hydrogen bonds and other interactions in the docked poses between the docked molecules and the list of important residues for either BACE-1 or AChE. The cutoff distances for hydrogen bonds and for VdW contacts were 3.5 Å and 5 Å, respectively. For BACE-1, docked molecules with at least four hydrogen bonds, including two hydrogen bonds with one or two catalytic Asp and at least eight contacts with the protein were considered “positively docked”. For AChE, molecules with all nine pre-determined important interactions were considered “positively docked”. The other criteria for decision making on molecular candidates are presented below.

### 2.3. Choice of Molecules by Weighted Scores Based on ISE Indexing and Docking Results

We obtained the scores of molecules from both our ligand-based ISE, as well as from docking. To decide on a final set of candidates, we needed to combine the scores from each molecule. That requires to produce “weights” for each of the scores.

In order to provide such weighted scores, we picked a test set made of 46 known AChE-BACE-1 dual inhibitors from the literature [30,33,69,70,71,72] and added 150 molecules picked randomly from ZINC, which were indexed by ISE and docked to both AChE and BACE-1 structures, similarly to our screened set of molecules (Supplementary Excel File, Table S10E). The score was adjusted so it would pick as many positives and leave out as many randoms. “Docking results” include the classification: docked/undocked (or 1/0, Supplementary Excel File, Table S10E) for each molecule, and the number of poses between 1–10, if it were docked (Supplementary Word File, Section 1.10).

**Figure 2:**
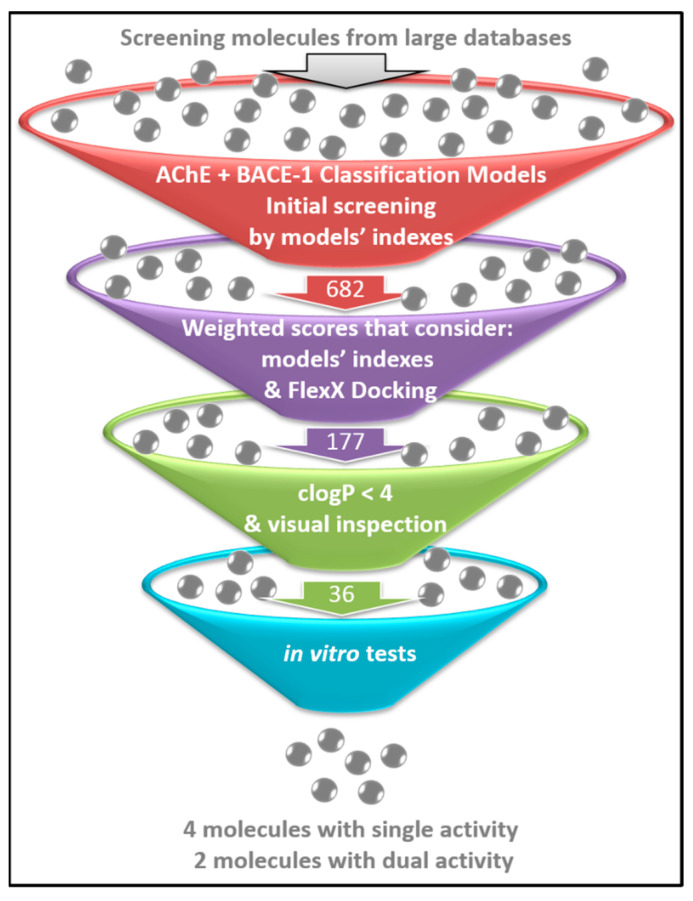
Research workflow: After the creation of ISE models for AChE and for BACE-1, large databases (Section 1.4) were screened by the models’ filters and 682 molecules had top scores in both. These molecules were docked by FlexX and were given weighted scores based on both the docking results and on ISE indexes (Supplementary Excel File, Table S10E). The 177 molecules with the highest weighted scores (Supplementary Excel File, Table S14E) were filtered again by calculated logP and visual inspection to obtain the final 66 molecules (Supplementary Excel File, Table S15E) of which 36 were purchased and sent for testing inhibitions of AChE and of BACE-1 (Supplementary Excel File, Table S16E). Six molecules inhibited either AChE or BACE-1, and two of them inhibited both enzymes (Supplementary Excel File, Table S17E). Details of the in vitro tests are given in Section 2.4 below and the results are presented in Supplementary Excel File, Tables S16E and S17E.

The ISE indexes and docking results for 680 molecules (the top scored ones) were used to construct a table combining the ligand based and structure based grades. Details are given in Supplementary Excel File, Table S10E and Supplementary Word File, Section 1.11. The choice of molecules was based on the weights of ISE scores and of docking, suggested by the test set described above. Molecules were selected if their weighted score >0.5 in all scores. Finally, we used the calculated logP (<4) and visually inspected the 2D structures of molecules to further eliminate molecules: 66 molecules were chosen for tests concerning the inhibition of AChE and BACE-1, but only 36 of those could be purchased (Supplementary Excel File, Tables S14E–S16E). The process of virtual screening is illustrated in Figure 2.

### 2.4. Biological Examination

#### 2.4.1. BACE-1 In Vitro Single Point Inhibition Measurement

Measurement of the proteolytic activity of BACE1 was based on the cleavage of the fluorogenic peptide substrate corresponding to the Swedish mutation of Alzheimer precursor protein (Mca-SEVNLDAEFRK(Dnp)RR. The fluorescence of 7-methoxycoumarin (Mca) is quenched by 2,4-Dinitrophenyl (Dnp) group in the uncleaved substrate, while after cleavage, the fluorescence of Mca is significantly increased. The reaction was performed in 24 µL volume in a black 384-well plate (Greiner Bio-One GmbH, Kremsmünster, 788075 Austria). Then, 8 µL of diluted inhibitor was pre-incubated with 8 µL enzyme solution for 30 min at 30 °C, the reaction started with the addition of 8 µL substrate. The reaction was executed for 2 h at 37 °C and the fluorescence was read at 0, 60 and 120 min. The fluorescence values were corrected using the starting values at 0 min. The assay buffer contained 1% DMSO in 100 mM sodium acetate, pH 4.0. The peptide substrate was purchased from R&D Systems (ES004), and we used a final concentration of 10 µM. The enzyme was also purchased from R&D Systems (931-AS-050) and used at the final concentration of 6.6 ng/µL. Fluorescence of Mca was detected at 320 nm excitation and 405 nm emission wavelengths. Single point inhibition measurements were conducted at 100 µM. Reaction conditions for BACE-1 inhibition measurements were used according to the supplier’s manual [73]. The reaction time and temperature were optimized, but the buffer composition and pH were used as written in the reference.

#### 2.4.2. BACE-1 IC_50_ Determination of Hits

Compounds inhibiting BACE1 activity by at least 50% were selected for dose response curve determination in a second round. For dose response measurements, a 3-fold dilution series containing 5 concentrations were used, starting from 100 µM as the highest concentration. Inhibition values were calculated from the fluorescence change of the 120 min reaction and normalized to the value of the uninhibited reaction. Results were fitted, and IC_50_ values were calculated using the Hill function of Origin 8.0 software. For validation of the inhibition, a known inhibitor, β-secretase Inhibitor IV (Merck 565788) was used; the determined IC_50_ value was identical to the literature data (15 nM) [74].

#### 2.4.3. AChE In Vitro Single Point Inhibition Measurement

Measurement of the enzymatic activity of acetylcholinesterase (AChE) was based on monitoring the increase in the yellow color (detected at 405 nm) of 5-thio-2-nitrobenzoate anion produced from thiocholine when it reacts immediately with a dithiobisnitrobenzoate (DTNB) ion. Acetylthiocholine was used as the substrate in the reaction, which is hydrolyzed by AChE to form the free sulfhydryl containing thiocholine. The reaction was performed in 150 µL in a clear, flat bottom, 96 well plate (Greiner655180). Then, 15 µL inhibitor, 10 µL enzyme and 5 µL DTNB (from a 10 mM solution) were added to 110 µL 100 mM sodium phosphate buffer, pH 8.0 and incubated for 10 min at 30 °C. After pre-incubation, we added 10 µL of substrate (to 500 µM final concentration). Absorbance was monitored continuously for 30 min at 30 °C. Absorbance change was calculated by correcting absorbance values using the starting absorbance. The recombinant human AChE enzyme was produced in stably expressing CHO-K1 cell line. The amount of enzyme required for an assay point was predetermined using a 2-fold dilution series to achieve approx. 0.8–1 linear absorbance unit change during the reaction time. Single point inhibition measurements were conducted at 100 µM concentration.

#### 2.4.4. AChE IC_50_ Determination of Hits

Compounds inhibiting AChE activity by at least 50% were selected for the second round, dose response measurements. In the dose response measurements, 7-point dilution series were used, a 3-fold dilution series with 100 µM as the highest concentration. Inhibition values were calculated from the absorbance change during the 30-min reaction and normalized to the value of the uninhibited reaction. Values were fitted, and IC_50_ values were calculated using the Hill function of Origin 8.0 software. For validation of the inhibition, a known inhibitor, donepezil, was used; the determined IC_50_ value was practically identical to the literature data (6.6 nM vs. 5.7 nM [75]). The assay condition was used for AChE as published [76]. The reaction time and temperature were optimized, but the buffer composition and pH were used as written in the reference.

#### 2.4.5. Animal Experiment

The animal-model protocol was carried out in accordance with “Guiding Principles for the Care and Use of Animals” based upon the Helsinki declaration and was approved by the Institutional Ethics Committee (permission number: 22.1/1268/3/2010). Six to seven months old male and female B6C3-Tg (APPswe, PSEN1dE9)85Dbo/J mice were used for the experiment. Animals were treated with the compound intraperitoneally daily for 14 consecutive days at the dose of 25 mg/kg. Each animal group was kept in a separate cage during the experiments. On each cage, there was an identification card with birth date, number and gender of animals. The injection of each substance was written on the identification card. Five to six animals per substance were used and identified by ear cutting. The tested compound was provided in powder formula. The planned experimental dose of the test compound was 25 mg/kg but since it was not water-soluble, the administration of it into the animal was not an exact dose.

#### 2.4.6. Detection of Amyloid Precursors by ELISA Method

Half brains of the animals were homogenized in 1 mL Tris buffer with 1% Triton X-100 and 2% inhibitor. The samples were centrifuged (21,000× *g*, 4 °C, 25 min) and the supernatant was retained (soluble fraction). The pellets (insoluble fraction) were homogenized with 70% formic acid and ultracentrifuged (100,000× *g*, 4 °C, 1 h). The supernatants were neutralized with Tris buffer. After that the supernatants and pellets of the first centrifugation were used as samples. Aβ40 and Aβ42 amyloid precursors were detected in brain homogenates by ELISA methods using kits (KHB3481 and KHB344, Life Technologies, 92008 Carlsbad, CA, USA) following the instructions of the manufacturer. Standard samples were: 500 pg/mL, 250 pg/mL, 125 pg/mL, 62.5 pg/mL, 31.25, 15.63 pg/mL and 0 pg/mL.

## 3. Results and Discussion

### 3.1. Comparison, Evaluation and the Best Physico-Chemical Properties of BACE-1 and AChE Models

We describe in this manuscript an effort to discover multi-targeted inhibitors of two enzymes with different mechanisms that are relevant to the blocking of Aβ42 by applying our unique, beyond state-of-the-art algorithm to ligand-based modeling for the two enzymes and combining it with our special approach to structure-based docking and to the analysis of docking results. Screening and scoring millions of molecules by the ligand-based models enables to pick only top scored molecules for subsequent docking. The analysis of docking also allows for prioritizing molecules, and combining these results with those of the ligand based screening was enabled by applying weights to each of the scores in a final score for each candidate. As far as we are aware, there are only a few previous studies that searched for single ligands of proteins from different families, which have very different catalytic mechanisms [77,78].

For BACE-1 inhibition—the AvI models were constructed with inhibitors having a wide range of IC_50_ values against sets of “inactives”: randomly picked molecules from the ZINC database (see Table 1). Four models were constructed, differing in the number of actives due to restrictions by Tanimoto similarity. All four models (1–4) had relatively high MCC values, close to 0.8, for their top filters (Table 1) and a high average AUC ca. 0.95, for the five-fold test sets. The selected model for screening from this group was model 2, which had both high MCC and AUC values. Model 1 had similar values for MCC and AUC as model 2, but model 2 was based on a more diverse set that was known to produce more diverse candidates by screening [79] The second type of BACE-1 models, HvL, compared the class of high activity molecules (IC_50_ < 100 nM) to lower activity molecules (IC_50_ > 1000 nM). The resulting MCC values for models 5–8 were 0.56–0.71 (Table 1), somewhat poorer than MCC values for the AvI models, because of greater similarity between the two classes, which makes distinguishing between classes a more difficult task. Interestingly, models with greater diversity (resulting in a smaller number of the more actives) had progressively better MCC values (but not AUC). One explanation is the ratio of high to low actives in each—it was 0.59 for model 5, 0.38 for model 6, 0.30 for model 7 and 0.24 for model 8. Model 8 was selected for screening also since it was expected to discover more diverse candidates by screening.

The filters of a model distinguish between classes: they are constructed of properties, and it is interesting to question which properties appear more among the many filters in each model and whether they could supply additional insights. Their appearances were counted in the filters of all five folds and presented in Table S4.

For the BACE-1 model 2, the prominent properties were subdivided surface area properties, e.g., SlogP_VSA3, SlogP_VSA0, as well as: 21 ≤ a_hyd ≤ 47 (numbers of non-polar atoms) and 416 ≤ weight ≤ 1176. In model 8, the prominent properties were partial charge properties and atom/bond counts properties: 1 ≤ chiral ≤ 8 (The number of chiral centers) and 3 ≤ a_nN ≤ 12 (Number of nitrogen atoms). Some of those properties also had relatively high MCC, e.g., a_hyd, weight (with average single MCC ca. 0.74) for model 2 and chiral, (with average single MCC 0.5) for model 8.

Five models were constructed for AChE inhibitors: one AvI and four HvL. The MCC for the filters of the AvI model (0.82) was higher—as expected—than any MCC for the HvL models, 0.65–0.71 (Table 2). Furthermore, high average AUC values were obtained for the validation sets: 0.97 for the AvI model and 0.78–0.88 for the HvL models (Table 2). The models chosen for screening were 1, 4 and 5 (colored gray in Table 2).

In the AChE AvI model 1, some of the best properties were: 21 ≤ a_hyd ≤ 40 (Number of non-polar atoms), a_nI = 0 (Number of iodine atoms), 1 ≤ FCharge ≤ 4 (Total charge of the molecule).

For HvL models 4 and 5 of AChE, the properties that appeared the most were: subdivided surface area properties and partial charge properties, (e.g., SLOGP_VSA4, BCUT_PEOE1) which were more elaborate than the properties that distinguish classes in the AvI model 1. For more data on MOE properties see Supplementary Excel File, Table S11E.

### 3.2. Results from Screening DrugBank

A previous version (4.0) of Drugbank [53] was used for screening. It included ~1400 FDA approved small molecule drugs and 5045 experimental (“discovery phase”) small molecules. Screening these molecules, despite the small size of that set, could be highly beneficial because molecules may be identified as candidates for different treatments than their original approval and could, thus, be suggested for repurposing.

We screened 1400 molecules from DrugBank-Approved and found only 107 molecules with a positive index for AChE model 1. Out of those, 33 molecules had positive indexes for model 2 of BACE-1 (Supplementary Excel File Table S7E). Several of those molecules are known as being targeted at muscarinic- and neuronal-acetylcholine receptors (e.g., terfenadine, darifenacin, tubocurarine, metocurine) and AChE (e.g., tubocurarine, irinotecan), (Table 3). Darifenacin was previously found as one of the best ten candidates for drug repurposing using docking methods [80]. All these molecules (except Irinotecan) were identified as negatives in the AChE HvL models 4 and 5.

Only two DrugBank-Approved drugs had positive indexes for all five models (Table 3): irinotecan (topoisomerase inhibitor) and aliskiren (renin inhibitor), while another one, nelfinavir (potent HIV-1 protease inhibitor), had positive indexes in four models. All the molecules that passed the previous two filtrations by ISE indexes (AChE model 1 index > 0, BACE-1 model 2 index > 0) were subjected to another filtration (detailed in Supplementary Excel File, Table S3E). As a result, only three approved drugs, indinavir, aliskiren and nelfinavir, were obtained and went through the docking procedure. They fulfilled the interactions conditions for docking BACE-1 but not AChE.

The next filtration was by combining ISE indexes and docking results through the weighted scores (Supplementary Excel File, Table S10E). That combination led to the passage of only two molecules: nelfinavir and aliskiren. After filtration with calculated logP, only aliskiren remained.

BACE-1 belongs to the aspartic protease family of proteins, which includes other targets of DrugBank-Approved ligands such as renin and HIV-protease. That may be the reason that drugs directed to those other aspartic proteases obtained higher indexes in the BACE-1 model (Table 3). Moreover, HIV protease inhibitors were already investigated for their effect on AD and some of them affected Aβ, though not directly through BACE-1 [81,82]. Renin inhibitor aliskiren was also found to be effective against Aβ toxicity [83].

In addition, eight molecules from DrugBank-Experimental passed the two ISE models as well as the docking filtration by weighted scores, but only six of them were successfully docked in both AChE and BACE-1 (Table 4 and Supplementary Excel File Table S18E), while two, DB02477 and DB02628, had high weighted scores despite the fact that they were not docked to AChE. Two of these six experimental drugs are stereoisomers targeting AChE: (R)- and (S)-tacrine(10)-hupyridone (codes in DrugBank: DB04614 and DB04615) and one (code in DrugBank: DB08749) targets BACE-1. This is an important validation of our models that “discover” annotated molecules that are not part of the training set. These DrugBank-Experimental molecules were compared by Tanimoto similarity index (TI) to the respective model training set; it was found to be structurally different. Specifically, for the AChE set, there were no molecules with TI similarity > 0.8 compared to DB04614/5, and for the BACE-1 set, there were no molecules with similarity TI > 0.7 compared to DB08749. These molecules appear in ChEMBL (CHEMBL76658 and CHEMBL244342) but not in our training sets. Eventually, only three out of the DrugBank-Experimental molecules reached the final list of 66 molecules but were not experimentally examined, as only 36 out of 66 could be purchased and sent for tests.

### 3.3. Selection of Best BACE-1 and AChE Structures for Docking

**Figure 3:**
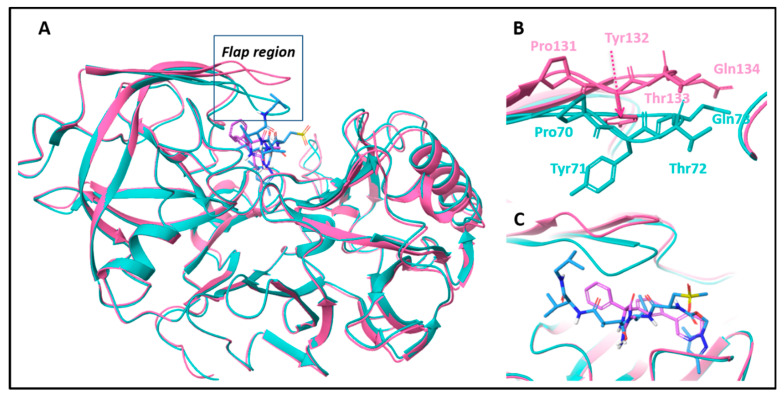
BACE-1 superimposed structures 2G94 (cyan) and 4DJW (pink) in closed and open conformations, respectively. (**A**) the whole enzyme. (**B**) An enlargement of the flap region with its residues that have the largest RMSD values (these are the same residues, but with different numbering in PDB structures 70–73 or 131–134). (**C**) ligands in the binding site: 2G94 peptidomimetic ligand in light blue, 4DJW aminohydantoin ligand in pink. The illustration was made with Maestro 11.0, Schrödinger.

To select structures of BACE-1 and AChE for docking, we compared 21 BACE-1 and 22 AChE structures from the Protein Data Bank in order to pick the most diverse ones (Supplementary Excel File, Tables S12E and S13E). Two BACE-1 structures, 2G94 and 4DJW, were found to be the most different among a group of superimposed BACE-1 structures according to an RMSD matrix. Both of these structures were aligned by pocket residues, where the residues with the highest RMSD (>2 Å) were Pro70, Tyr71, Thr72 and Gln73. Indeed, these residues are included in the “flap” region of different BACE-1 structures (Figure 3, Figures S9 and S10) [85,86] The two types of flap conformations in BACE-1 crystal structures are called “closed flap” and “open flap”. A link between the length of these conformations and the molecular weights of ligands that block the catalytic machinery of BACE-1 was suggested [87]. The catalytic residues are nearer in space to the closed conformation and more distant from the open one. Specifically, distance ranges of 8–10 Å for the “closed flap” are associated with molecular weights range of ligands MW = 400–1000 Da. For distance ranges of 11–16 Å of the “open flap”, ligands with of molecular weights 130–430 Da were found [87] Indeed, the peptide–mimetic ligand crystallized with BACE-1 in the “closed flap” structure 2G94 [88] is large, whereas the aminohydantoin ligand in the “open flap” structure 4DJW [89] is much smaller and not peptide-like.

Another publication suggested some additional representative structures for BACE-1 that we avoided due to their low resolution or the presence of mutations or missing residues or lack of proper ligands: those are structures 1FKN, 1TQF, 1W51 and 1XN3 [85,90] In addition, all these structures were similar regarding the flap region, while 4DJW (published in 2012 [89]), used by us, differs from that group significantly. The choice between 2G94 or 4DJW for screening was based on the ability of these structures to discriminate between stronger and weaker inhibitors by docking molecules that were tested in vitro. The test set of 48 BACE-1 highly active molecules (max. activity of IC_50_ 100 nM) and 125 BACE-1 molecules with low activity (minimal IC_50_ of 1000 nM) had no similar molecules with TI greater than 0.7. Of the 48 highly active molecules, 17 were successfully docked to 4DJW and only 5 were docked well to 2G94. Of the 125 low activity ligands, 21 were docked to 4DJW and 57 were docked to 2G94. All of the docking experiments were performed on the “protonated” configurations in both structures, with Asp32 protonated and Asp228 ionized. The TP/FP for 4DJW is, thus, ~2.1, while for 2G94 it is only 0.23. Therefore, it was decided to continue with 4DJW for docking the screened databases molecules (see Table S1 and Section 1.7 in the Supplementary Word File).

All aspartic proteinases use the “double pronged” pair of aspartates for the catalytic cleavage of peptide bonds. The bell-shaped pH profile of the catalytic activity in aspartic proteinases is due to the lack of catalytic activity in low pH (both aspartates are protonated) and a similar lack of activity in high pH (both aspartates are deprotonated). The pH of maximal activity differs in that family due to cellular location and to electrostatic effects by neighbor residues, but it is clear that top activity requires one protonated and one deprotonated carboxylate group of the two Asps [91]. Either Asp32 or Asp228 (or in BACE-1, alternative numbering: Asp93 and Asp289, see Table S3 in Supplementary Word File) are protonated and the other is ionized, for full catalytic activity. We tested the effect of the protonated and deprotonated states of Asp32 in docking to both 4DJW and 2G94 and found a minor effect on docking results. In 4DJW, we compared the docking in two protonation states (Figure S6). In the first structure, Asp32 deprotonated structure, 207 molecules were docked (out of the total of 680), while in the second structure, Asp32 protonated structure, 265 molecules were docked, and in the third structure, an Asp32 protonated structure as well but with protonation/deprotonation of other residues and flip conformational modifications—277 molecules were docked (Supplementary Word File, Section 1.9). Overall, 101 molecules were successfully docked in all three structures of BACE-1. A molecule was considered docked if it was docked in at least one out of three structures.

For selecting the most appropriate crystal structure of AChE we docked to three structures: 4EY7, 4M0F and 4M0E. Here, we used known positives and negatives. AChE PAS inhibitors (“positives”) were docked together with a set of molecules picked randomly from the ZINC database (“negatives”). The structure of 4M0F was found to have the highest specificity (by identifying the true negatives better than others), having a specificity of 76% compared to 62% and 50% for 4M0E and 4EY7, respectively (Table S2). This enzyme structure had a few additional advantages over other structures: a high (2.3 Å) resolution, complexed with a small molecule (territerm B MW~450 Da) and bound to the PAS as well as to the deep “gorge” leading to the catalytic site. It is also a human enzyme with sequence differences compared to the much more abundant crystal structures of the Torpedo Californica or of mouse [62]. For the docking of the 680 molecules see Methods Section 2.2.5 and Supplementary Word File, Section 1.10.

### 3.4. In Vitro Activity of Hits and In Vivo Results of a Dual Hit

**Figure 4:**
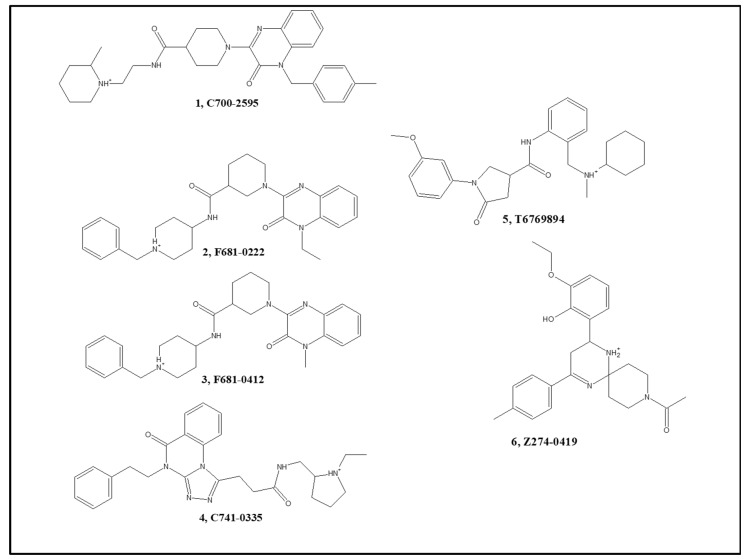
The six hit compounds.

Out of 36 molecules sent for testing on AChE and BACE-1 enzymes, two nearly identical molecules (F681-0222 and F681-0412) inhibited both targets, where AChE IC_50_ was <10 μM and BACE-1 IC_50_ was ~60 μM for both (Figure 4 and Table 5). Additionally, two molecules were active only on AChE (with IC_50_ < 10 μM) and two molecules were active only on BACE-1 (with >50% inhibition of BACE-1 activity at 100 μM). Overall, four molecules were active on each of the two enzymes, two of the four inhibiting both. Activity results in percent inhibition of AChE and BACE-1 for the rest of the tested molecules that were found to be inactive are shown in Table S5.

**Figure 5:**
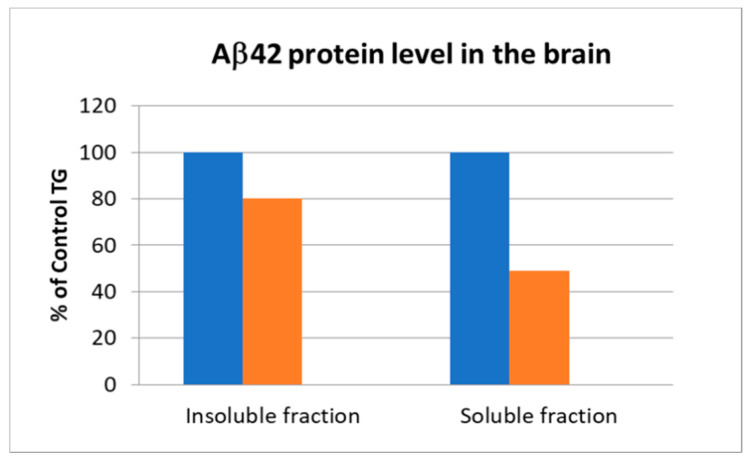
Decrease in Aβ42 level in the brains of mice. In blue—control TG = transgene mice; In orange—tested compound, 2.

Aβ42 protein in the brain homogenates was detected by ELISA method. Treatment of animals with compound 2, F681-0222, for 14 days at a dose of 25 mg/kg/day decreased the secretion of Aβ42 amyloid precursor in the insoluble fraction and even more in the soluble fraction (Figure 5). While compound 2 showed significant reduction in the soluble Aβ42 in brain tissue, it had minimal effect on the Aβ40 soluble fraction (see Supplementary Excel File, Table S19E).

### 3.5. New Scaffolds for AChE/BACE-1 Inhibitors

Three of the hits, 1–3, contained a quinoxalinone moiety and one hit, 4, had a closely related (fused) quinazolinone moiety. The literature search on quinoxalinone in AChE and BACE-1 inhibitors gave no results, but the quinazolinone fragment was found in some AChE inhibitors [92] and was mentioned in a patent for BACE-1 inhibitors (US 2013/0150387 A1) [93], while functionalized quinoxalinones were considered privileged scaffolds [94]. However, some similar quinoxaline derivatives were found to inhibit Butyrylcholinesterase [95]. Structurally, most of the AChE active molecules (Table 5, 1–3) are more similar among themselves than the BACE-1 actives group (Table 5, 5–6). The two molecules found to have dual activity (2 and 3) differed only by a methyl group and had a Tanimoto similarity index of 0.98 (Table S6). Molecule 1 is relatively similar to 2 and 3 with TI around 0.7, and all these three molecules differ from molecule 4 (that inhibits AChE) with TI values of 0.3–0.38. The BACE-1 inhibitors 5–6 are even more diverse in structure with a TI value of 0.3 between them and even smaller TI values in comparison to 1–4, and to 6, being somewhat larger for 5. The six new inhibitors are more different by Tanimoto similarity than the actives of AChE and BACE-1 that produced the models. Values of TI were mostly less than 0.6, and none had TI > 0.7. The six inhibitors of Figure 4, thus, present new scaffolds.

### 3.6. Analysis of Computational vs. Experimental Results

The model indexes and docking results of the AChE active molecules 1–4 (Table 6) provide a strong connection between experimental and computational results. All four molecules have an index of 0.78 for AvI model and indexes >0.37 for the two HvL models used for screening, with three of them having indexes >0.6 in both HvL. All four molecules were docked to AChE structure with at least two useful poses out of 10, of which two molecules had eight or nine poses out of ten. However, five tested molecules (out of 36) that followed these requirements as well, were not active on AChE (False positives), e.g., molecule 5.

In the screening of molecules by our ligand-based ISE models, we focused on molecules that had positive indexes. The six active molecules (Table 6) indeed had positive indexes in all five models mentioned, of which two were AvI models (AChE model 1 and BACE-1 model 2) and the rest were HvL models.

Based on the results of Table 6, would it be possible to predict which molecule is a stronger inhibitor of one of the two enzymes? Most of the AvI results are better for AChE than for BACE-1 (average 0.74 for AChE, only 0.25 for BACE-1). Of all six molecules, only the BACE-1 active molecule 6 had a better index for BACE-1 than for AChE. Judging by AvI models alone suggests that molecules 1–5 should be better inhibitors of AChE than of BACE-1. That is indeed borne out for molecules 1–4, as presented in Table 5. Results of the HvL models were quite similar for AChE (models 4 and 5) and BACE-1 model 8. However, for molecule 6, both AvI and HvL indexes were larger for BACE-1 than for AChE, again in agreement with the experimental results (Table 5). The last two columns of Table 6 present the numbers of docked poses, which were superior for AChE compared to BACE-1. There were overall 35 docked poses to AChE PAS and 22 docked poses for the same molecules in theBACE-1 catalytic site. The difference is much more prominent because the docked poses are counted for a single structure of AChE but for three structures of BACE-1. However, it is again molecule 6 that has more docking poses for BACE-1 than AChE. It is also interesting to find that molecule 2 has many more docking poses than molecule 3, despite the very small structural difference between the two.

Overall, the much larger AvI indexes for AChE models may suggest that affinities are greater to AChE than to BACE-1, which we find in Table 5. However, we do not anticipate an ability to use indexes of molecules in a specific model to suggest which of them has a greater affinity: molecule 5 (Table 6) has the largest AvI index value for AChE-1 but no affinity was detected experimentally.

It may be of interest to apply cutoff indexes and docking results from the six active molecules in order to select molecules with larger probability for success. Applying the AChE and BACE-1 parameters (Supplementary Excel File, Table S20E) to the 36 tested molecules, we obtain all the active molecules and additional few false positives for AChE (five molecules) but a greater number of false positives for BACE-1 (13 molecules). We may conclude that the AChE models are better than the BACE-1 models in separating actives and inactives, and that for BACE-1, the HvL model provides better separation than its AvI model.

### 3.7. Examination of Docking Poses in AChE and BACE-1

**Figure 6:**
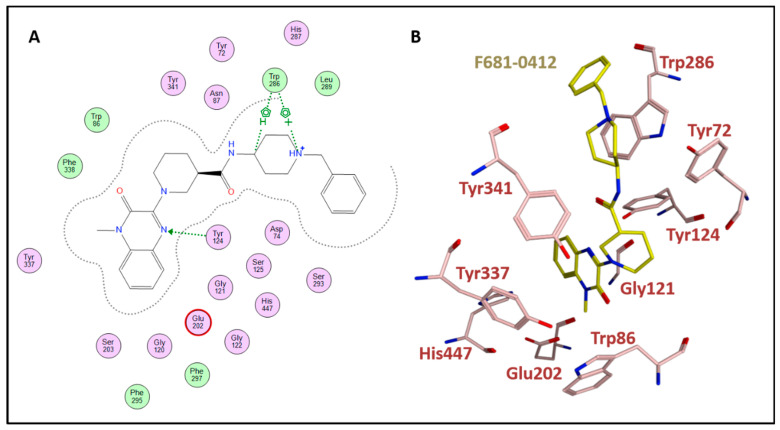
(**A**) The interactions of the dual hit, 3, (F681-0412), with binding site residues in AChE (created with MOE2011). All the important AChE residues are present and in close contacts with the ligand. Non-polar residues are colored green, polar residues are colored light purple, acidic residues are further annotated by a red ring. A hydrogen bond is marked with an arrow, and aromatic hydrocarbon interactions with H atom or a cation are marked as well. In addition to the H-bonds shown by arrows, the other residues interact with the ligand by VdW atom–atom interactions. Most of the residues mentioned in Section 2.2.5 and Supplementary Word File, Section 1.10, are shown in this figure. (**B**) A corresponding 3D pose of hit 3.

The two bifunctional hits contain a fragment of donepezil, which is a known, approved AChE inhibitor: a benzyl-piperidine fragment. However, while the benzyl ring of this fragment occupies the CAS (catalytic region part of the AChE gorge), it interacts with the PAS (upper part of the gorge) in the docked poses of molecules 2 and 3 (Figure 6 and Figure S12). According to Cheung et al., [62] the interactions of donepezil with AChE in the crystal structure (PDB code: 4EY7) are π-stacking of the benzyl with Trp86 in the CAS, the aromatic indanone ring stacks with Trp286 in the PAS and the piperidine ring forms a hydrogen bond through water with Tyr337 and Tyr341. Here, in the docking results of 2 and 3, the benzyl-piperidine occupied the PAS while the quinoxalinone ring was closer to CAS and interacted with residues in PAS as well. Similarly, molecules 1 and 4 were also docked in a manner where the charged nitrogen of piperidine or pyrrolidine was docked in the PAS and an aromatic fragment of the molecules was in the CAS (for more docking poses in AChE see Figures S11–S13).

Regarding the benzyl-piperidine moiety of donepezil, it occupies less than a third of the molecular weight of the two successful dual inhibitors 2 and 3. Additionally, the donepezil-AChE crystal structure contains a water molecule that forms hydrogen bonds with AChE residues and with donepezil. Similarly, AChE bound to CPT-11 also includes the participation of a water molecule in the interactions [96], while water molecules were not a part of our docking procedure for the molecular hits. Another docking study of AChE inhibitors that contain quinazolinones also found that the positively charged amine part of the molecules was docked in the CAS region, while the fused ring part of the molecules was docked in the PAS [97].

**Figure 7:**
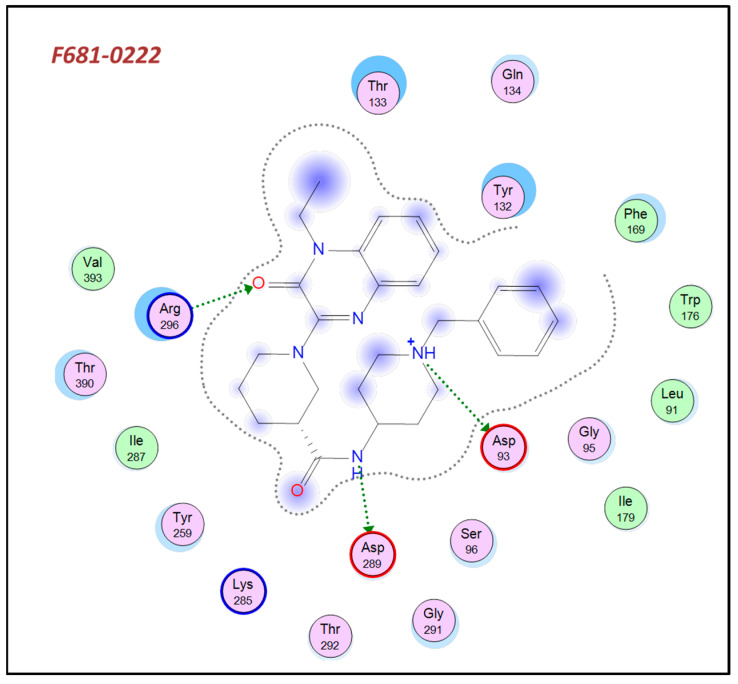
The interactions of hit molecule 2 with binding site residues of BACE-1 Asp93 deprotonated structure. Both of the catalytic aspartates form hydrogen bonds with the hit molecule.

When examining the dual hits 2, 3, which were docked to the deprotonated Asp93 BACE-1structure, both Asp93 and Asp289 catalytic residues form hydrogen bonds with the protonated nitrogen on the piperidine ring and the adjacent amide nitrogen, respectively (Figure 7 and Figure S14). Other hydrogen bonds with 2 and 3 are between the carbonyl oxygen of the quinaxolinone and Arg296, in addition to a hydrogen bond that was formed in 3 between the quinaxolinone’s nitrogen and Thr292 hydroxyl.

Hit molecule 5 was docked in the two protonated BACE-1 structures with a total number of five poses. Hydrogen bonds are between Gly291 carbonyl and the exocyclic amide nitrogen and between Asp93 and the carbonyl of the pyrrolidone ring (Figure S15). In one of the poses, Gly291 carbonyl forms another hydrogen bond with the protonated nitrogen. Hit molecule 6 was docked in all BACE-1 structures, with six poses in two protonated structures and two poses in the deprotonated structure. In the protonated structure, the phenolic hydroxyl formed a hydrogen bond with Asp93 and the protonated nitrogen with Gly95. In the deprotonated Asp93 structure, there were other hydrogen bonds for the protonated nitrogen with Gly291, Thr292 and Asp289 (Figure S16).

### 3.8. Multi-Targeting Finds Different Affinities for Different Targets

The IC_50_ values for AChE (~4 µM) and BACE-1 (>60 µM) are very different: We could expect that based on BACE-1 inhibition, the currently dual candidates would turn out to be useless. However, it is clear that one of the two candidates shows also inhibition in vivo, lowering by about 50% the level of the AD culprit Aβ42. This may be a demonstration of the idea that was promoted by several multi-targeting publications [98,99], e.g., that dual action by a single molecule does not require that the molecule should have similar affinities to both targets.

## 4. Conclusions

We applied our computational methods in order to search for dual-inhibitors that could prevent the production of hazardous Aβ42 peptides and avoid their aggregation. In order to do that, we combined our in-house ligand-based modeling by ISE with our method of docking and of docking analysis. Millions of molecules were screened and scored by ISE models in order to reduce the large numbers and to pick the top ones for docking to chosen crystal structures of AChE and of BACE-1.

We applied our ISE algorithm to produce several AvI and HvL models for blocking AChE PAS and BACE-1. Top scored molecules were subsequently docked to the PAS of AChE and to the catalytic site of BACE-1. We used known dual inhibitors in order to decide upon the relative weights of ligand-based and of structure-based results for the final scoring of each molecule.

Screening by our ligand based models provided candidates that have different scaffolds from the set of active molecules that we learned from. We achieved “scaffold hopping”, which is an important ingredient for novelty and a potential basis for further structural modifications. In docking the candidates to both enzymes, many more poses were found to dock well to the PAS of AChE than to the three mono-anionic states of BACE-1. The so called “Peripheral Anionic Site”, indeed, includes no anionic residues but a large set of aromatic residues that supply negatively charged aromatic rings. The “docking advantage” of the overall neutral PAS over the negatively charged BACE-1 site may help to understand the immense synaptic turnover by AChE, as huge numbers of positively charged (quaternary ammonium) acetylcholine are “docked” to the enzyme on the membrane external surface due to the negative charge supplied by residues with aromatic side chains Phe Trp, Tyr and His.

We combined those computational methods in order to pick only a few candidates that could supply dual action simultaneously. The chance to find such candidates even from screening a few million molecules is not large, and only two molecules achieved that goal, out of the screening of ~3 million. However, that number is not remote from expectations in high-throughput screening experiments. If the chance to find a single effective candidate is 1 in 1000, then we should multiply that number by 1:1000 to learn the chance of finding a single dual-active molecule, which is 1 in a million. We found two out of 3 million, which is within the error deviations.

Multi-targeting for targets of different protein families by computational methods is rare compared to multi-targeting proteins in the same protein family, such as Src and Abl kinases [100]. We achieved multi-targeting for AChE and BACE-1 by subsequent screening with ISE classification models and docking in both of the proteins’ crystal structures. Novel diverse inhibitors, which target both AChE and BACE-1, were confirmed in vitro, (the best two compounds: AChE: IC_50_= 4–7 µM, BACE-1: IC_50_ = 50–65 µM). One dual inhibitor also showed activity in vivo by reducing Aβ42 levels by 20–50 percent in mice. These molecules can serve as promising lead compounds in drug development for Alzheimer’s disease.

Some molecules that docked well to BACE-1 were inactive in vitro. Some of them docked to multiple BACE-1 structures and with many poses. We conclude that our docking filters for BACE-1 actives should be improved.

Another observation for dual inhibitor screening optimization is that the molecules with the highest weighed scores (based on indexes and docking parameters for both targets) were not the ones with activity on any of the targets. This might result mostly from relatively low indexes for the BACE-1 AvI model, which contributed a major part to the weighted scores.

## Figures and Tables

**Table 1:** Comparison of BACE-1 ISE models’ properties and performance.

Model Type	Actives vs. Randoms (AvI)				High Actives vs. Low Actives (HvL)			
Model No.	1	2 ^a^	3	4	5	6	7	8 ^a^
**Tanimoto**	1.0	0.9	0.8	0.7	1.0	0.9	0.8	0.7
**Actives** **IC_50_ (nM) cutoff**	<10,000	<10,000	<10,000	<10,000	<100	<100	<100	<100
**no. Actives**	1316	617	344	194	438	177	93	51
**Inactives** **IC_50_ (nM) cutoff**	randoms	randoms	randoms	randoms	>1000	>1000	>1000	>1000
**no. Inactives**	46,743	46,743	46,743	46,743	747	463	307	214
**Average ^b^ best filter MCC**	0.79	0.79	0.76	0.76	0.56	0.60	0.66	0.71
**Average ^b^ AUC**	0.95	0.95	0.95	0.94	0.82	0.78	0.83	0.77

**^a^** The chosen models for screening are colored in grey; ^b^ average of 5 folds.

**Table 2:** Comparison of AChE ISE models’ properties and performance.

Model Type	Actives vs. Randoms (AvI)	High Actives vs. Low Actives (HvL)			
Model No.	1 ^a^	2	3	4 ^a^	5 ^a^
**Tanimoto**	1.0	1.0	0.8	0.9	0.9
**Actives** **IC_50_ (nM) cutoff**	<10,000	<100	<100	<100	<100
**no. Actives**	428	211	65	109	109
**Inactives** **IC_50_ (nM) cutoff**	randoms	>1000	>1000	>1000	>3000
**no. Inactives**	37,467	197	65	112	86
**Average ^b^ best filter MCC**	0.82	0.65	0.71	0.69	0.71
**Average ^b^ AUC**	0.97	0.88	0.78	0.86	0.86

**^a^** Chosen models for screening are colored in grey; ^b^ average of 5 folds.

**Table 3:** Potentially repurposed drugs from DrugBank-Approved.

Drug Name (DrugBank 4.0 [53] Name)	Target(s) ^a^	AChE Indexes			BACE-1 Indexes	
		Model 1	Model 4	Model 5	Model 2	Model 8
**Indinavir (DB00224)**	HIV-1 protease	0.51	−0.47	−0.52	0.85	0.83
**Saquinavir (DB01232)**	HIV-1 protease	0.40	−0.39	−0.40	0.85	0.44
**Aliskiren (DB01258)**	Renin	0.73	0.19	0.20	0.83	0.81
**Nelfinavir (DB00220)**	HIV-1 protease	0.87	0.00	0.12	0.83	0.10
**Terfenadine (DB00342)**	M-ACh-R ^b^	0.67	−0.20	−0.40	0.81	−0.75
**Darifenacin (DB00496)**	M-ACh-R	0.88	−0.47	−0.68	0.58	−0.31
**Tubocurarine (DB01199)**	AChE, N-ACh-R^c^	0.54	−0.51	−0.41	0.58	−0.68
**Metocurine (DB01336)**	N-ACh-R, M-ACh-R	0.54	−0.38	−0.44	0.27	−0.76
**Irinotecan (DB00762)**	DNA- topoisomerase, cholinesterase AChE [84]	0.33	0.26	0.14	0.18	0.65

^a^ Targets from DrugBank website-partial list ^b^ M-ACh-R–muscarinic acetylcholine receptors; ^c^ N-ACh-R–neuronal acetylcholine receptor subunit alpha2.

**Table 4:** Six experimental drugs with high models’ indexes and docking results.

DrugBank Name	AChE Indexes			BACE-1 Indexes		AChE Poses ^a^	BACE-1 Poses ^b^	Target ^c^
	Model 1	Model 4	Model 5	Model 2	Model 8			
**DB01721**	0.33	−0.46	−0.66	0.85	0.75	6	6	Gag-Pol polyprotein
**DB02009**	0.84	−0.45	−0.66	0.85	0.79	4	27	Gag-Pol polyprotein
**DB04424**	0.78	−0.73	−0.83	0.58	0.66	4	16	Trypsin-1
**DB04614**	0.88	0.46	0.39	0.85	0.09	9	2	AChE
**DB04615**	0.88	0.46	0.39	0.85	0.09	8	7	AChE
**DB08749**	0.85	0.53	0.34	0.75	−0.15	3	30	BACE-1

^a^ The maximal no. of poses in AChE is 10; ^b^ The sum of poses for 3 BACE-1 structures, so the maximum number of poses for BACE-1 is 30. ^c^ Targets from DrugBank website.

**Table 5:** Hit compounds on AChE and/or BACE-1.

Cmp. No.	Cmp. Name	AChE % Inhibition at 10 µM	AChE IC_50_ (µM)	BACE-1 % Inhibition at 100 µM	BACE-1 IC_50_ (µM)
**1**	C700-2595	65	8.2	19	n.m. ^a^
**2**	F681-0222	64	4.4	69	63
**3**	F681-0412	36	6.7	66	55
**4**	C741-0335	76	9.7	6	n.m.
**5**	T6769894 ^b^	19	n.m.	82	60
**6**	Z274-0419	−12	n.m.	76	150

^a^ n.m. not measured; ^b^ Molecule 5 is the only one from Enamine, while the rest are from ChemDiv database.

**Table 6:** Models indexes and docking results for the six hit molecules.

Cmp. No.	AChE Indexes			BACE-1 Indexes		AChE Poses ^a^	BACE-1 Poses ^b^
	Model 5	Model 4	Model 1	Model 8	Model 2		
**1**	0.63	0.62	0.78	0.58	0.18	8	1 (1)
**2**	0.6	0.62	0.78	0.56	0.18	9	2 (1)
**3**	0.61	0.63	0.78	0.54	0.18	2	4 (2)
**4**	0.37	0.44	0.78	0.64	0.18	4	2 (1)
**5**	0.6	0.77	0.8	0.57	0.16	8	5 (2)
**6**	0.09	0.13	0.49	0.59	0.6	2	8 (3)

^a^ One crystal structure of AChE was used for screening by docking so the maximum no. of poses in AChE is 10; ^b^ For BACE-1, three similar crystal structures were used for docking, so the maximum no. of poses in BACE-1 is 30 (see Supplementary Word File, Section 1.9) The number in parentheses is the number of structures where the molecule was docked—out of 3.

## Data Availability

All relevant data are included in the supplementary word file and excel file.

## Supplementary Materials

The following supporting information can be downloaded at: https://www.mdpi.com/article/10.3390/ijms232113098/s1.

## Abbreviations

AD	Alzheimer’s disease
Aβ	Amyloid-β
APP	Amyloid precursor protein
AChE	Acetylcholinesterase
BACE-1	Beta-site APP cleaving enzyme 1 (β-secretase)
NMDA	N-methyl D-aspartate
MTDL	Multi-target directed ligand
PAS	Peripheral anionic site
ISE	Iterative stochastic elimination
MCC	Matthews’s correlation coefficient
AUC	Area under curve
ROC	Receiver operating characteristic
TI	Tanimoto similarity index
