# Supplementary material for: Dual Inhibitors of AChE and BACE-1 for Reducing Aβ in Alzheimer’s Disease: From In Silico to In Vivo
Source: Int J Mol Sci. 2022 Oct 28;23(21):13098. doi: 10.3390/ijms232113098 (PMC9655245; doi:10.3390/ijms232113098)
Supplement: Supplementary file 1 [file ijms-23-13098-s001.zip › Supplementary Material TEXT.pdf]

Supplementary Material

AChE and BACE-1 Dual Inhibitors for Reducing Aβ in Alzheimer's Disease: From *In-Silico* to *In-Vivo*

Noa Stern[1], Alexandra Gacs[2], Enikő Tátrai[2,3], Beáta Flachner [5], István Hajdú [5,6], Krisztina Dobi[5], István Bágyi[5], György Dormán[5], Zsolt Lőrincz[5], Sándor Cseh[5], Attila Kígyós[3], József Tóvári[3,4], and Amiram Goldblum[1,]*

[1]*Molecular Modeling and Drug Design Lab, Institute for Drug Research, The Hebrew University of Jerusalem, Israel*

[2]*Department of Experimental Pharmacology, National Institute of Oncology, Budapest, Hungary*

[3]*KINETO Lab Ltd, Budapest, Hungary*

[4]*Department of Tumor Biology, National Korányi Institute of TB and Pulmonology, Budapest, Hungary*

[5]*TargetEx Ltd, Dunakeszi, Hungary*

[6]*Institute of Enzymology, Research Centre for Natural Sciences, Hungarian Academy of Sciences, Budapest, Hungary*

* Correspondence: amiramg@ekmd.huji.ac.il

Introduction

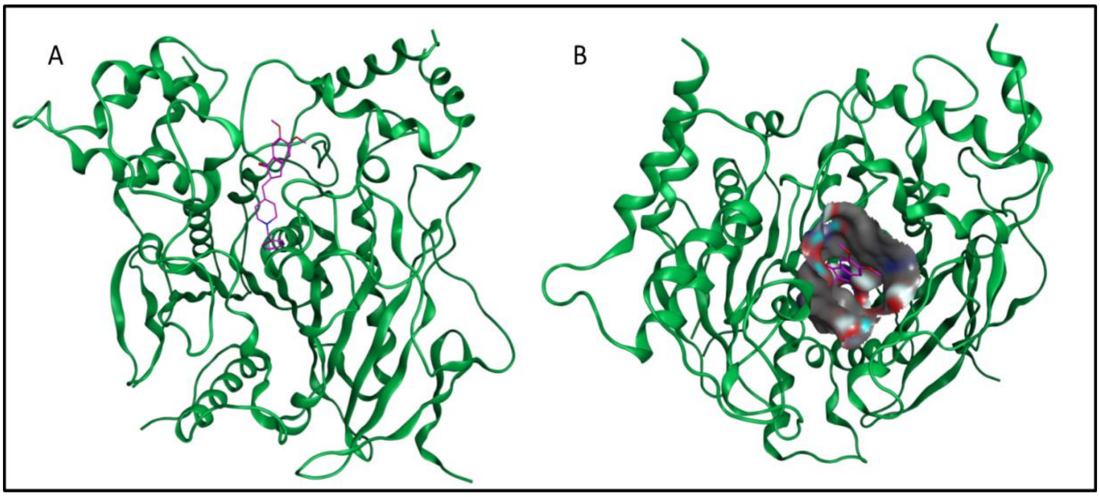

Figure S1. Views of acetylcholinesterase (AChE) binding site in one of its the crystal structures (PDB entry: 4EY7) viewed by MOE2011. AChE in green and the ligand, Donepezil, in magenta. (**A**) A view from the side. (**B**) A view from the top to the pocket entrance, showing the pocket surface. The PAS, peripheral anionic site, is located on the entrance to the pocket.

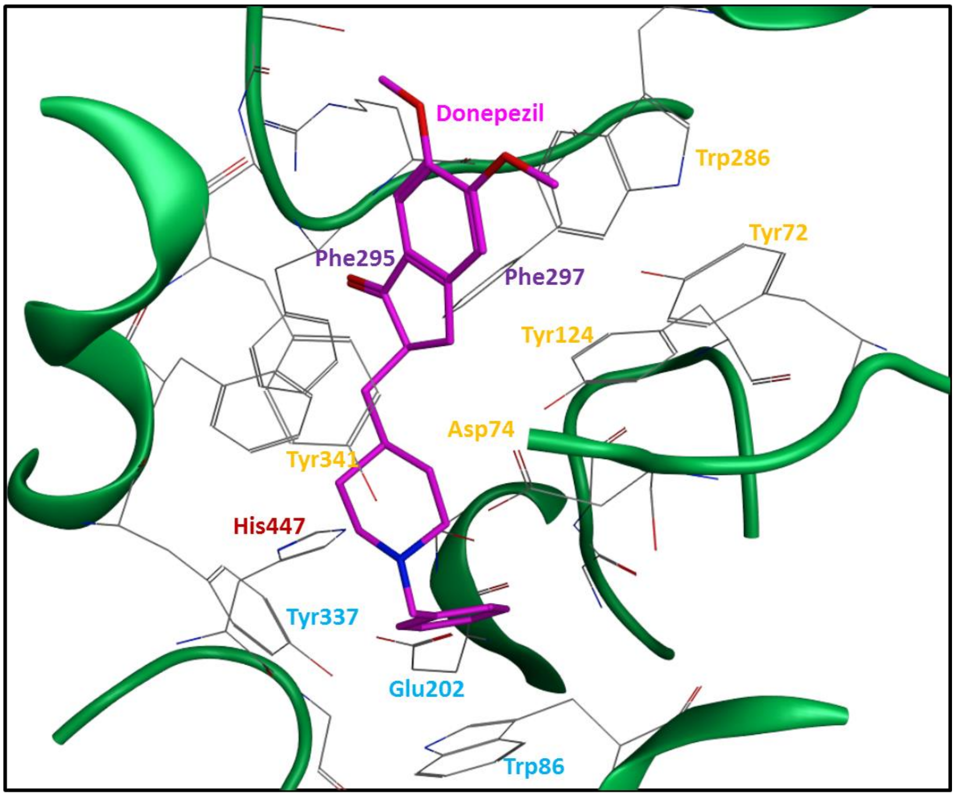

Figure S2. A View on AChE binding site residues in one of its crystal structures (PDB entry: 4EY7) viewed by MOE2011. AChE in green, its residues in grey and the ligand, Donepezil, in magenta. His447 of the catalytic triad (in red); CAS (catalytic site) residues: Trp86, Tyr337, Glu202 (in light blue); Acyl pocket residues: Phe295, Phe297 (in purple); PAS residues: Tyr72, Asp74, Tyr124, Trp286, Tyr341 (in yellow). While the CAS is on the "bottom" of the AChE binding pocket, the PAS is on the "top".

Methods

1.1 Datasets Initial Preparation and Filtration

Data was obtained from CHEMBL (2014 version) for acetylcholinesterase (Homo sapiens), TARGET_CHEMBLID 220. The chosen activity was IC_{50} since it had the largest set of values, 4252. Many molecules were removed: molecules with no SMILES, with activity units other than nM, with no IC_{50}, with validity comment "outside typical range" or "potential transcription error", with any operator other than "=" (i.e. > or <) and molecules with IC_{50} >50,000 nM. Only molecules with "assay organism" = Homo sapiens were kept. Then, by excel text search, molecules that contain phosphorous atom or carbamate functionality in their SMILES strings were removed, since they belong to irreversible AChE inhibitors group (inhibitors that bind to AChE covalently).[1] Name duplicates were removed as well, where for an identical pair of molecules with different activity values, the molecule with higher IC_{50} (lower affinity) was kept while the other was erased.

Afterwards, manual inspection of the molecules was carried out and molecules with potential to form covalent bond with AChE were removed (carbamates and some esters). In addition, only PAS binders (known or potential ones) were kept. The choice of PAS potential binders was based on the literature sources mentioned in CHEMBL. A molecule was kept in the list if it was designed in a paper to be PAS binder (sometimes with docking in AChE structure) or had a similar structure to known PAS binders (e.g. donepezil): usually include at least two rings/ring systems, aromatic and/or with nitrogen atom, attached by a linker to create a long molecule that fits the AChE gorge and may form interactions with its many aromatic residues by $\pi-\pi$ or π-cation interactions.

Then, Tanimoto similarity filtration was performed in the range of 0.8-1.0. SMILES duplicates were removed by Tanimoto filtration. A list of 428 AChE molecules was obtained after Tanimoto = 1.0 and removal of molecules with very low activity, IC_{50} > 10,000 (Supplementary Material: Excel file).

For BACE-1 (Homo sapiens), data was downloaded from CHEMBL (2012 version) TARGET_CHEMBLID 4822. The chosen activity was IC_{50} since it had the largest set of values, 2731. Duplicates of molecules' names and SMILES were removed, as well as entries with the operator ">" and entries with no activity values. For an identical pair

of molecules with different activity values, the molecule with higher IC_{50} (lower activity) was kept, while the other was erased. Then, Tanimoto similarity filtration was performed in the range of 0.7-1.0.

1.2 Preparation of the Training Sets

MOE (Molecular operating environment, 2011 version)[2] wash option was used to prepare all the active and inactive molecules (including removal of group I metal ions, deprotonation of strong acids and protonation of strong bases), and then partial charges were given and 2D descriptors were calculated. Four of the 2D descriptors, Lipinski's properties: lip_don, lip_acc, logP and weight, were used to find the "applicability domain" of AChE and BACE-1 datasets, in order to filter molecules from ZINC database[3] that were used as randoms, assumed inactive molecules, for part of the models. This filtering of randoms according to applicability domain was meant to form a set of inactive molecules similar in those four properties to the set of active molecules. This action would hopefully lead to formation of better model filters.

Specifically, about 48,000 ZINC molecules were filtered according to the following applicability domain ranges calculated for AChE: lip_acc 2-9, lip_don 0-5, logP 1.51-9.56, weight 227-705 gr/mol and for BACE-1: lip_acc 0-18, lip_don 0-9, logP -0.36-8.25, weight 144-900 gr/mol. The applicability domain values were determined from calculation of average descriptors' values ±2 standard deviations, of each descriptor, for each dataset.

Then, data was inserted to KNIME software,[4] where active and inactive classifications were given to the molecules, according to IC_{50} values, by receiving values of 1 or 0. Next, the descriptors were filtered in case they had low variance (value 0.0) and if they had high correlation ($r^2 > 0.81$) based on a correlation values matrix. The resulting combined list of actives and inactives, including their descriptors and classification, was divided randomly to five groups, that each contains the same number of actives and inactives. Every four groups made a training set and the remaining group was used as a test set. The input for ISE algorithm, therefore, included five training files with 80% of the data and five test files with 20% of the data (each).

1.3 Partition to "Actives" and "Inactives" in Two Types of Models

Data for the first BACE-1 model type included randoms from ZINC as inactives, and actives with a cutoff of IC_{50} = 10,000 nM (in manuscript, Table 1 models **1-4**). Data for

the second BACE-1 model type ("high vs. low" or HvL) included high activity molecules with IC_{50} values smaller than 100 nM versus molecules with low activity on the target with IC_{50} > 1000 nM (Table 1 models **5-8**). Data for the first AChE model included randoms from ZINC as inactives, and actives with cutoff of IC_{50} = 10,000 nM (in manuscript, Table 2 model **1**). Data for the second type of AChE models included high activity molecules with IC_{50} values smaller than 100 nM versus low activity molecules IC_{50} > 1000 nM (in manuscript, Table 2 models **2-4**). HvL model (model **5** in Table 2) with Tanimoto = 0.9 filtration was constructed with the same molecules as in model **4** but with a larger gap between the "high" and "low" groups with inactives having IC_{50} > 3000 nM. The best of all the models, for each target, was used for screening (see manuscript Results section). All types of models are summarized in Figure S3.

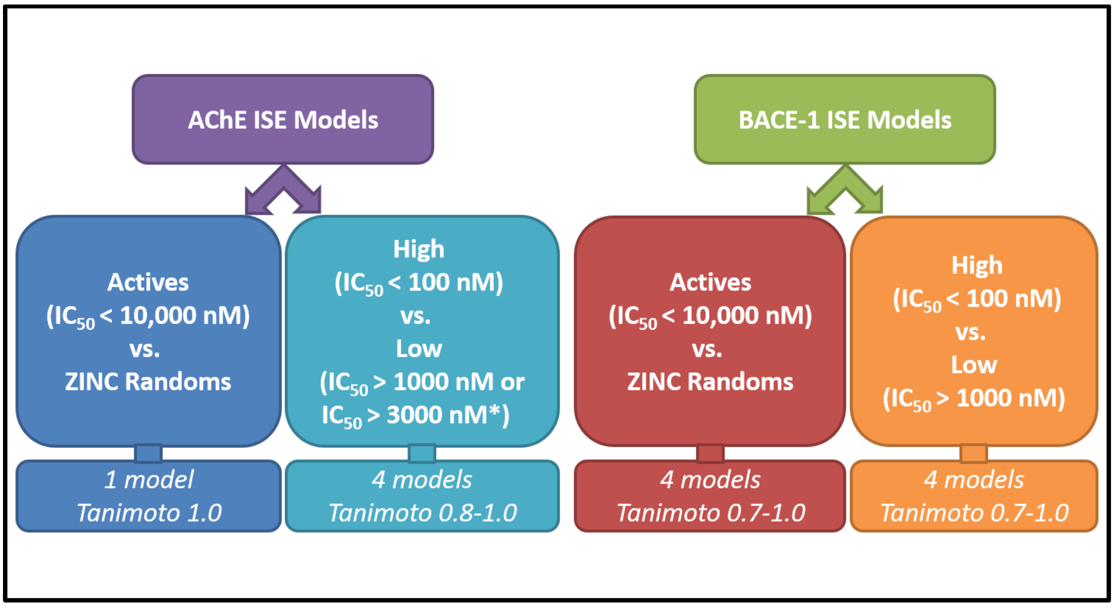

Figure S3. The various ISE models built. Not all were used for screening (see Results section). *Model **5** of AChE has IC_{50} > 3000 nM cutoff for selection of low-activity molecules.

1.4 ISE Method

ISE (Iterative Stochastic Elimination) algorithm was created to solve complex problems with multiple variables, which are dependent on each other, and each variable has many possible values.[5] At first, ISE was used to solve problems related to interactions with proteins and structures of proteins. For instance, if a stable conformation in a docking process is required, the dihedral angles that define the conformation are the variables that have many possible values. Later, ISE was also used for building various

classification models that can distinguish between types of molecules (generally can be called "positives" and "negatives"), e.g., actives and inactives on a protein target, soluble in water versus insoluble etc. In the case of using ISE for classification, the variables are molecular properties (descriptors) that have different ranges (values) for the "positives" and "negatives", where the latter usually stand for actives and inactives. In both applications of ISE, random values of variables are sampled and with the help of a scoring function (e.g., free energy for structural problems or MCC – Matthew's correlation coefficient) their contribution to a high or low scores is measured. Then, the worst variables' values are eliminated and the others continue to another random sample creation and evaluation until a certain number of combinations' threshold is reached. Here, with the help of ISE, classification models for two protein targets, AChE and BACE-1, were constructed. Each model is made of a set of descriptor ranges filters.

1.5 ISE Output and Models Validation

An ISE script for filters generation was run for each of the five training sets and then the obtained filters were used for each model to score its corresponding test set. ISE output for each of the five models included a filters file. All the five filter files were combined to form the filters file of the final model, which was, later, used for screening of unknown compounds. Additionally, five files with ISE indexes for the five test sets' molecules were obtained- which allowed model validation, as will be explained bellow. First, models were evaluated by the filters MCC values, giving some indication of the separation between TP, NP, FP, and FN (where T- true, F-false, N-negatives or inactives, P-positives or actives). MCC ranges from -1 to 1, where a value of 1 indicates the perfect prediction, value of 0 on a random prediction and a value of -1 on a contradiction between prediction and observation. Second, five test sets were used to evaluate the five models, each one created from 80% of the data. The molecules were scored using the filters of each of the five models and AUC (area under curve) of ROC (receiver operating characteristic) curves were obtained.

1.6 Screening External Databases with ISE Models

The external databases (e.g., Enamine, ChemDiv) molecules were prepared with washing, partial charge calculation and 2D descriptors calculation in MOE. Then, the molecules were filtered by the selected models' filter files to obtain indexes. First, only

molecules with positive indexes for AChE model **1** were picked. Second, a filtration with the other AChE models **4, 5** and BACE-1 models **2, 8** was performed for these picked molecules simultaneously. The selected molecules for subsequent docking had to follow a condition: at least four positive indexes, including for AChE model **1**, for one of the AChE models **4, 5** and for both BACE-1 models. For the Drugbank molecules, the condition for selecting molecules was reduced and molecules with positive indexes for only AChE and BACE-1 models of actives vs randoms (AChE model **1** and BACE-1 model **2**) were selected.

Specifically, Enamine database was first screened with AChE model **1** filters to obtain 36,394 molecules with positive index values. Next, the selected molecules were filtered by AChE models **4** and **5** and by BACE-1 models **2** and **8** and 665 molecules were selected. A similar procedure was used for the other databases. For ChemDiv database 38,171 molecules passed the first filtration by AChE model **1** and 1805 molecules were selected after the filtration with the other models. Filtration of natural products databases with AChE model **1** resulted in 8422 molecules with a positive index, including 1594 molecules from Princeton NP, 3415 from IBS-NP and 3413 from Analyticon NP. The second filtration of natural products, by other models, resulted in 675 molecules. From Drugbank 33 approved molecules and 46 experimental molecules were selected after two filtrations (see Supplementary Excel file).

The 3224 selected molecules from all the mentioned databases were gathered and filtered again by more strict conditions, in order to reduce the number of molecules that would be filtered by docking. This smaller group of molecules must follow at least one of the following conditions. The first condition is that a molecule should have AChE model **1** index > 0.5, as well as for BACE-1 model **2**. The second condition is that BACE-1 model **8** index should be > 0.5 as well as one of the other AChE models, **4** or **5**. Filtration with another condition (in parallel), which was an index combination (index of AChE model **1** + index of BACE-1 model **2** + 30% of the larger index of AChE model **4** or **5** + 70% of BACE-1 model **8** index), and then joining the lists of molecules and removal of duplicates resulted in 682 molecules that continued to the docking stage (see Supplementary Excel file with the selected molecules and their indexes).

1.7 Selection of a Crystal Structure for Docking in BACE-1

The interactions of BACE-1 and its ligands in crystal structures were examined by looking at binding data from PDBSUM.[6] A list of hydrogen bonds and hydrophobic interactions between BACE-1 and its ligands was made for 75 crystal structures from PDB.[7] Then, 21 structures were chosen out of the above list (some of them from the same research groups) according to the following criteria: the structure had to include a ligand, high resolution < 2Å, no mutations and no missing segments.

The PDB codes of the chosen structures are: 4DJU, 4DJV, 4DJW, 4DJX, 4DJY, 3LPJ, 3LPK, 3L58, 3L5E, 3L5F, 3KMX, 3KMY, 3CIB, 3CIC, 3CID, 2QMG, 2QMD, 2QMF, 2QP8, 2G94, 1XN2. Those remaining 21 structures were superimposed with MOE software and a pairwise RMSD matrix was calculated (for Cα atoms of all residues), Figure S4. Average RMSD for Cα atoms and for pocket residues, both were < 1Å. The structures were divided into two groups according to largest RMSD difference and two different structures: 2G94 and 4DJW, one from each group, were chosen for docking a test set of known BACE-1 ligands with low and high IC_{50} values in order to select one structure for screening.

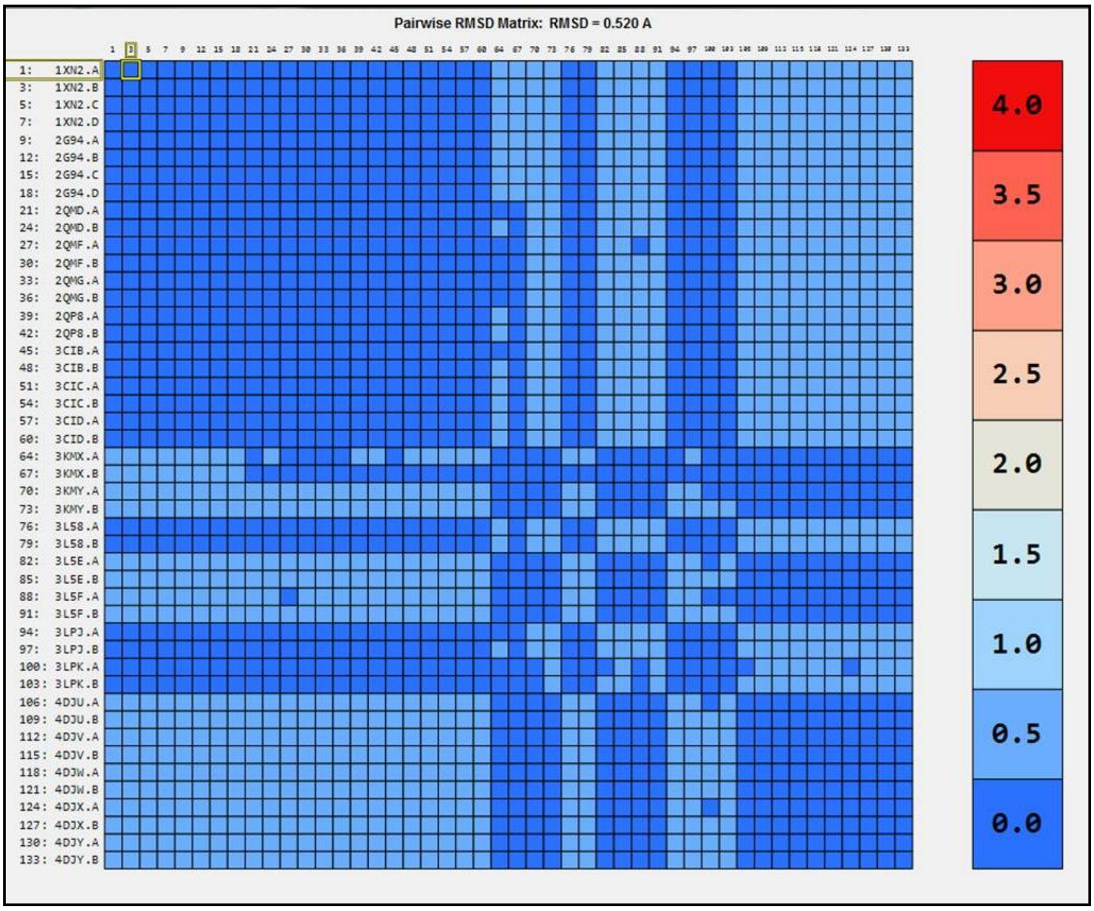

Figure S4. A pairwise RMSD matrix, by MOE superimposition of BACE-1 21 crystal structures (and their different chains). The RMSD differences are very small (< 1 Å) for all the structures, but still they can be divided into 2 groups according to the size of RMSD.

Therefore, a set of molecules with 48 BACE-1 inhibitors with higher activity (IC_{50} < 10,000 nM) and 125 inhibitors with BACE-1 lower activity (IC_{50} > 10,000 nM) was docked to the two chosen crystal structures, each with the option of Asp32 protonated and deprotonated so each molecule was docked 4 times (see explanation for protonation states in structure preparation, 1.9). Specifically, the list of test molecules was turned from SMILES (after washing, including pronation/deprotonation by MOE) to .oeb format (input file format for FRED[8] docking), that included 200 conformations for each molecule, with OMEGA[9] software. Molecules with undefined stereochemical centers were given stereochemistry with *flipper* command, creating stereo-isomers for many molecules. The tested crystal structure, 4DJW or 2G94, was inserted to the MAKE RECEPTOR in OEDocking 3.0.0 (Openeye). No constrains were applied and all options were chosen as default, except the size of box which was enlarged. The resulting conformations library and obtained .oeb structure files were used as input for docking with FRED. For each molecule, 30 poses were calculated by FRED, and the rest of the parameters were the default. Molecules that had several stereoisomers were considered docked if at least one of its isomers was docked. Docking results analysis and choice of best poses will be described in 1.10. The chosen structure for screening was the one that separated better the high- from low-affinity ligands (Table S1).

Table S1. Docking results for the test set molecules by FRED to two BACE-1 structures in two protonation states of Asp32 (in %)

BACE-1 Structure	%TP	%FP
4DJW protonated	35	17
4DJW deprotonated	52	17
2G94 protonated	10	46
2G94 deprotonated	15	49

1.8 Selection of a Crystal Structure for Docking in AChE

Out of 170 crystal structures of AChE structures in the PDB, only seven were of "Homo sapiens only". Out of those structures one had low resolution 3.2 Å and one structure had no ligand, which left five structures to choose from: 4M0E, 4M0F[10], 4EY5 (with huperazine A), 4EY6 (with galantamine) and 4EY7 (with donepezil).[11] According to MOE superimposition of the five structures (including their A and B units) RMSD was 0.305Å (for Cα atoms of all residues) and 0.202 Å just for A units of the structures.
Since a structure with a ligand that binds to the PAS was preferred, it left 4M0E/F and 4EY7. The resolution of these structures was < 2.5 Å and they had no mutations. However, these structures had two breaks of six amino acids and three amino acids between Pro258-Asn265 and Asp494-Pro498, respectively.
Despite these chain breaks in AChE human structures, it was decided to use them for docking and not use the mouse structure, which was whole, since the breaks (filled by structure preparation of MOE) were far from the binding site, Figure S5. In addition, there could be important differences between the mouse and human structure, that have a sequence identity 88%, according to BLAST[12], similar to differences that were discovered between human and torpedo structures (with sequence identity of 56%, according to BLAST).[11]

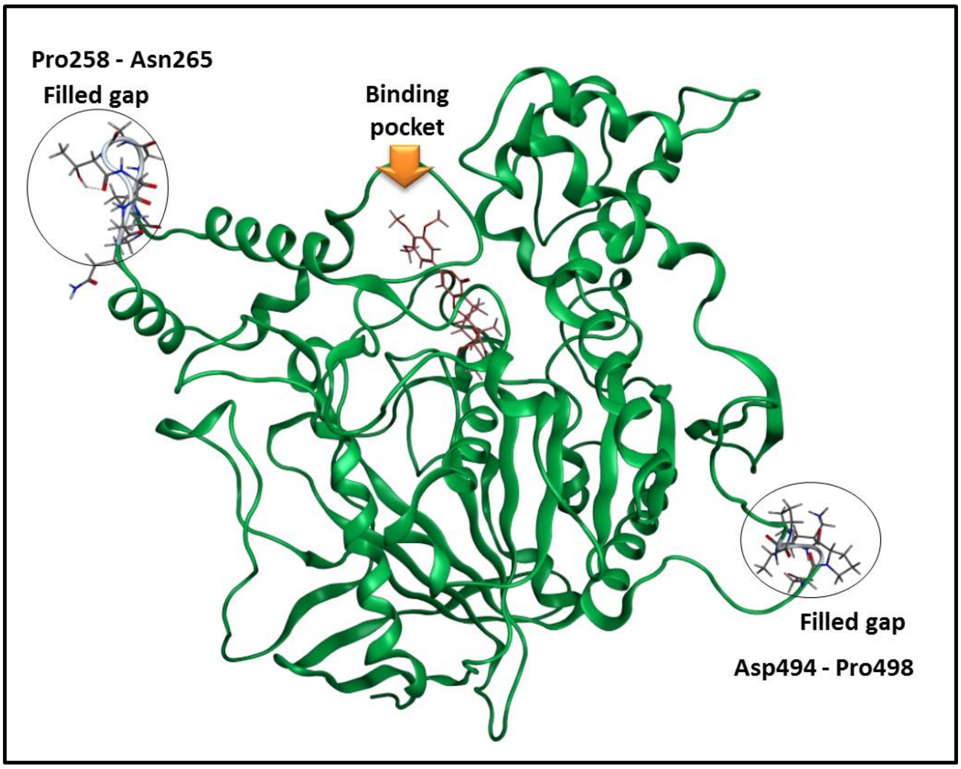

Figure S5. AChE structure with its gaps filled by MOE structure preparation. Both of the gaps were far from the binding site and therefore the structure was used for docking.

The final choice between the three AChE structures was based on a test set that was docked by FlexX and included 10 AChE active- and diverse molecules (with Tanimoto value 0.7) and 100 ZINC random molecules. The chosen structure had the highest specificity value (the ability to identify TNs- "true negative" molecules, Equation S1), though with lower sensitivity value (the ability to identify TPs- "true positive" molecules, Equation S2) Table S2. This test also helped in determination of the number of hydrophobic interactions needed for a molecule to be considered as docked in AChE (for explanation-see 1.10). The number of interactions that led to a higher specificity value was chosen.

Equation S1:

$$specificity = \frac{TN}{TN + FP}$$

Equation S2:

$$sensitivity = \frac{TP}{TP + FN}$$

Table S2. Docking results of the test set molecules in three AChE crystal structures: Specificity (%TN) and Sensitivity (%TP)

	4EY7	4M0E	4M0F
**%TN**	50	62	76
**%TP**	80	70	50

1.9 Crystal Structures Preparation for Docking

We decided not to include a water molecule in the binding site, since water is replaced by the inhibitor molecule,[13] though binding of ligand to BACE-1 through water was reported before.[13-15] In addition, the decision about the protonation state of the catalytic Aspartates was based on several papers that investigated the different options and concluded that there can be several preferred states, depending on ligand type.[16, 17] The options that repeated the most were one protonation on Asp32 or Asp228 (on the inner oxygen atom, Figure S6) or di-deprotonated state. However, other options were suggested as well.[18] Therefore, before screening large databases by docking, we

decided to examine the docking of known BACE-1 active and inactive molecules in two BACE-1 structures: one state with catalytic Aspartic acids totally deprotonated and another with one protonation, and the choice was protonation on Asp32 (see about the effect of protonation in Table S1).

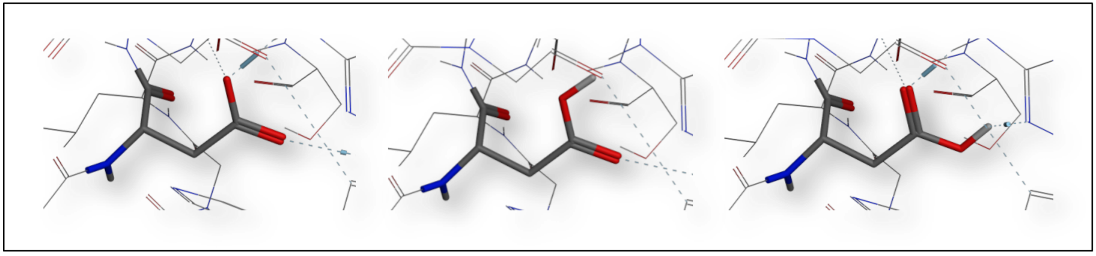

Figure S6. 3 possible protonation states possible for Asp32 in BACE-1 (MOE). Left: di-deprotonated state (one of the chosen positions in this work). Middle: mono-protonated, one proton "out". Right: mono-protonated, one proton "in" (one of the chosen positions in this work).

PBD structures, 4DJW and 2G94, were downloaded from PDB site. Unwanted chains, water molecules and ligands were removed with MOE (except for the ligand in the binding site) and the protonation state for catalytic Asp32 was determined as deprotonated or protonated for each structure. Only the catalytic Aspartates were treated among the protein residues. MOE structure preparation menu was used for the protonation and other structure issues needed handling (adding hydrogen atoms, capping N and C termini).

Regarding the selected structure for databases screening, 4DJW (chosen over 2G94, see Table S1), both previous structures, protonated and deprotonated, were used in addition to another protonated structure that was modified from the protonated structure file, by protonate 3D protocol of MOE, which determines ionization states of acidic and basic residues, tautomers of His, Asp, Glu and flips of His, Asn and Gln residues. To sum up, three 4DJW structures with different protonation states were used for screening external databases.

For AChE, PBD structures were downloaded from PDB site (PDB codes: 4EY7, 4M0F, 4M0E). Unwanted chains, water molecules and ligands were removed with MOE, except for the ligand in the binding site. MOE structure preparation menu was used for the addition of hydrogen atoms, capping N and C termini and filling the chain breaks. The ionization state of Asp74, Glu202, Glu292, Arg296, Glu450 and His447 was determined as neutral, while Glu334 was negatively charged. In addition, His447 was manually flipped so it would be in a position for making a hydrogen bond with Glu334 as they are part of the catalytic triad. This was also the reason for their protonations

states' choice (neutral and negative, respectively, as mentioned above). The other mentioned residues were examined because they are in the binding cavity or were mentioned as important in previous research.[19] Then, protonation was performed with the protocol Protonate 3D. The above list of residues was set as "protected" so their protonation state would remain during the calculation. The resulting structures were saved as PDB files and used for docking in FlexX.

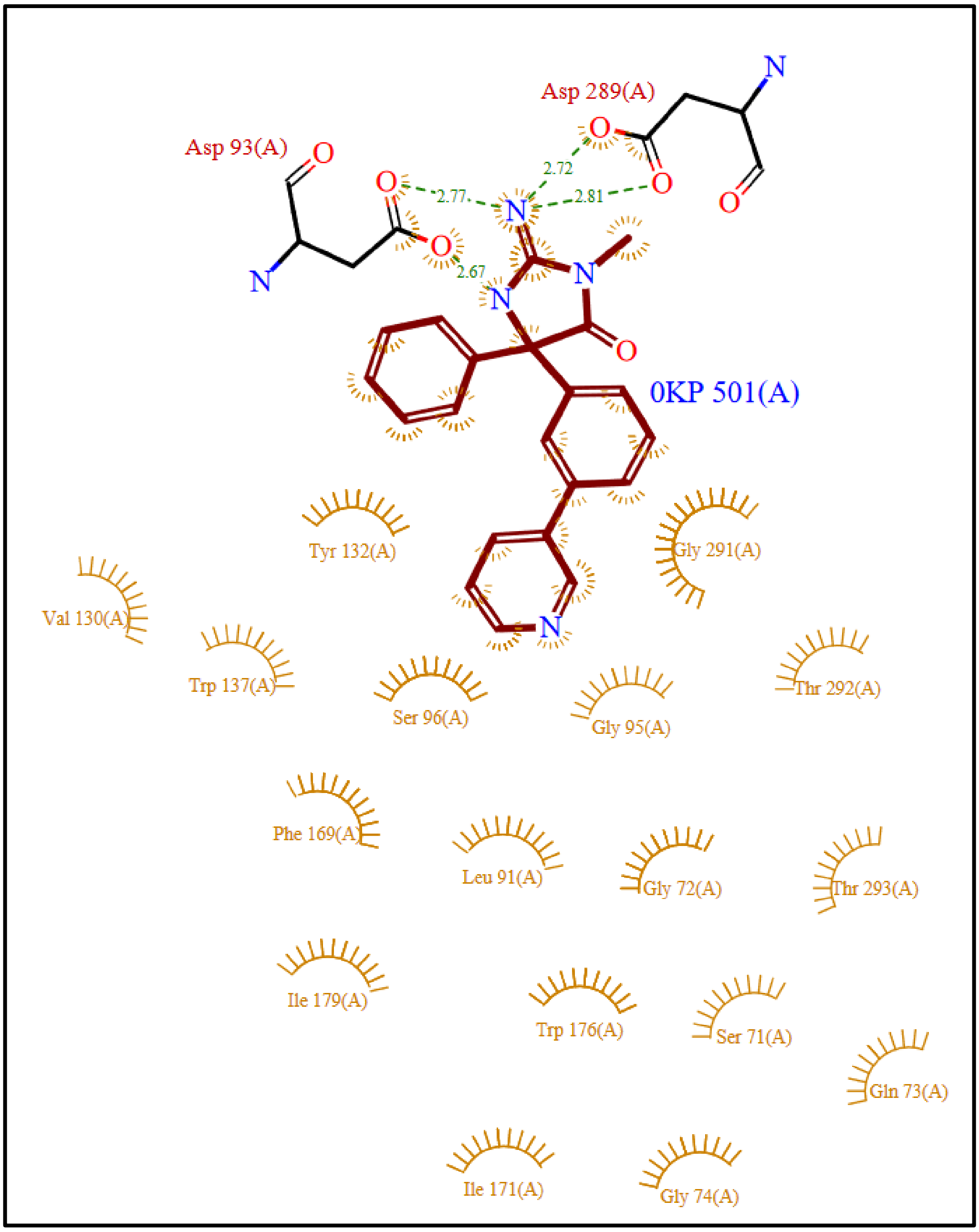

Figure S7. Interactions of BACE-1 with a ligand (0KP, in brown) from PDBSUM (PDB entry: 4DJW). Residues that form VdW interactions with the ligand are colored in light-brown. Hydrogen bonds are marked by green dashed lines with the residues that are black colored. For BACE-1 alternative residues' numbering, see Table S3.

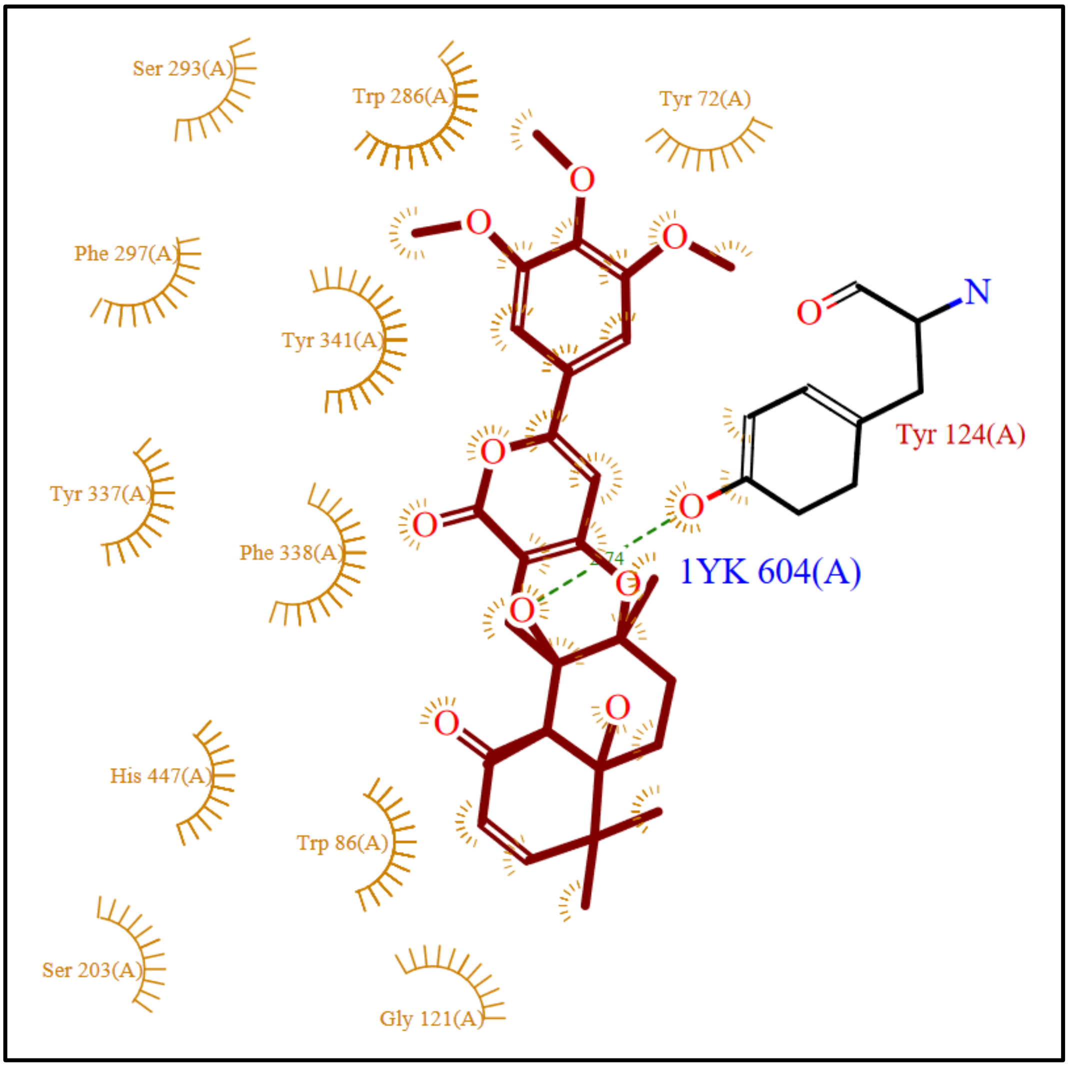

Figure S8. Interactions of AChE with a ligand (1YK, in brown) from PDBSUM (PDB entry: 4M0F). Residues that form VdW interactions with the ligand are colored in light-brown. Hydrogen bonds are marked by green dashed lines with the residues that are black colored.

1.10 Docking and Results Analysis

FlexX was used for docking molecular databases (e.g., Enamine, ChemDiv) in AChE and BACE-1 structures. It was also used before for AChE structure selection using a test set of known active molecules and randoms. However, for BACE-1 structure selection a test set that was docked by FRED docking. The structure input file was imported to FlexX after MOE structural preparations. The last structure modifications of choosing important residues and protonation adjustment were done in FlexX. Specifically, important residues were selected by default in FlexX and other known residues, unselected by FlexX, were added to the list based on previous inspection of crystal structures. For example, the ligand in 4M0E was located at the top of the gorge, which led to a default selection of only the top gorge residues, so bottom gorge residues

had to be added manually. Protonation was adjusted according to the file imported from MOE. The library of molecules to dock was prepared in MOE by washing, determining partial charges and minimization. For each molecule 10 poses were created. The parameters were all default.

The best docked poses were not selected by docking scores, but according to the existence of important interactions of the ligand to the protein structure (AChE or BACE-1). Therefore, the hydrogen bonding and hydrophobic interactions of residues to ligands were counted in the selected 21 PDB BACE-1 structures and in 19 PDB AChE structures, according to PDBSUM, and the residues with interactions that appeared in most of the crystal structures were picked as "important residues". Important interactions repeated in many BACE-1 structures are listed in Table S3. We comment that in several structures the numbering of residues was different, e.g., the catalytic Aspartates were numbered 93 and 289.

For AChE, only hydrophobic interactions repeated many times in the various structures (including Torpedo Californica, mouse and human structures). Some residues created hydrogen bonding occasionally, but since they created hydrophobic interactions most of the times, they were not considered as hydrogen bonding residues. (e.g., Glu202, His447). The residues that created hydrophobic interactions in most of AChE crystal structures were (numbering according to human structure): Tyr72, Trp86, Gly121, Tyr124, Glu202, Trp286, Tyr337, Tyr341 and His447.

Table S3. Important hydrophobic interactions and H-bonding in BACE-1 crystal structures

Hydrophobic Interacting Residues	Hydrogen Bonding Residues	Alternative Numbering	Atoms Interacting
Gly11	Asp32 (catalytic)	93	OD1, OD2
Leu30	Gly34	95	O
Ser35	Thr72	133	N, OG1
Tyr71	Gln73	134	N
Phe108	Asp228(catalytic)	289	OD1, OD2

Thr231	Gly230	291	O
	Thr232	293	N, OG1

Results and Discussion

Table S4: The best descriptors and their appearance (in % and number) in the selected AChE and BACE-1 models

Model No.	Target	Descriptor	%in Model	No. Filters
**1**	AChE	a_hyd	70.73	609
		vsa_acid	60.63	522
		a_nI	53.89	464
		GCUT_SMR_0	49.01	422
		FCharge	48.32	416
		a_ICM	32.29	278
		BCUT_PEOE_0	23.23	200
		a_nBr	21.84	188
		PEOE_VSA-3	20.56	177
		SMR_VSA1	16.38	141
**4**	AChE	SlogP_VSA4	40.29	591
		mutagenic	36.67	538
		PC-	27.33	401
		BCUT_PEOE_1	26.45	388
		b_rotR	25.56	375
		SlogP_VSA7	21.88	321
		SlogP_VSA8	20.65	303
		GCUT_SLOGP_3	19.84	291
		SMR_VSA0	17.72	260
		logS	17.52	257
**5**	AChE	SlogP_VSA7	40.97	449
		BCUT_PEOE_1	34.12	374
		SlogP_VSA4	30.38	333
		SlogP_VSA8	25.27	277

		SMR_VSA0	21.08	231
		VDistEq	20.07	220
		GCUT_SLOGP_3	19.80	217
		SlogP_VSA1	19.25	211
		BCUT_SLOGP_2	18.80	206
		Q_VSA_NEG	17.24	189
**2**	BACE-1	SlogP_VSA3	57.04	948
		Weight	50.90	846
		GCUT_SLOGP_3	47.41	788
		SlogP_VSA0	37.18	618
		a_hyd	34.84	579
		BCUT_SLOGP_1	32.91	547
		GCUT_SMR_1	23.29	387
		weinerPol	22.38	372
		Q_VSA_FPPOS	21.12	351
		BCUT_SLOGP_3	21.06	350
**8**	BACE-1	a_nN	46.62	828
		SlogP_VSA5	39.86	708
		chiral	34.74	617
		SlogP_VSA4	34.35	610
		GCUT_SLOGP_0	32.15	571
		BCUT_SLOGP_3	29.84	530
		Q_VSA_PPOS	27.93	496
		b_double	19.88	353
		a_nBr	18.92	336
		SMR_VSA5	18.64	331

For more data on MOE descriptors see *https://www.chemcomp.com/journal/descr.htm*

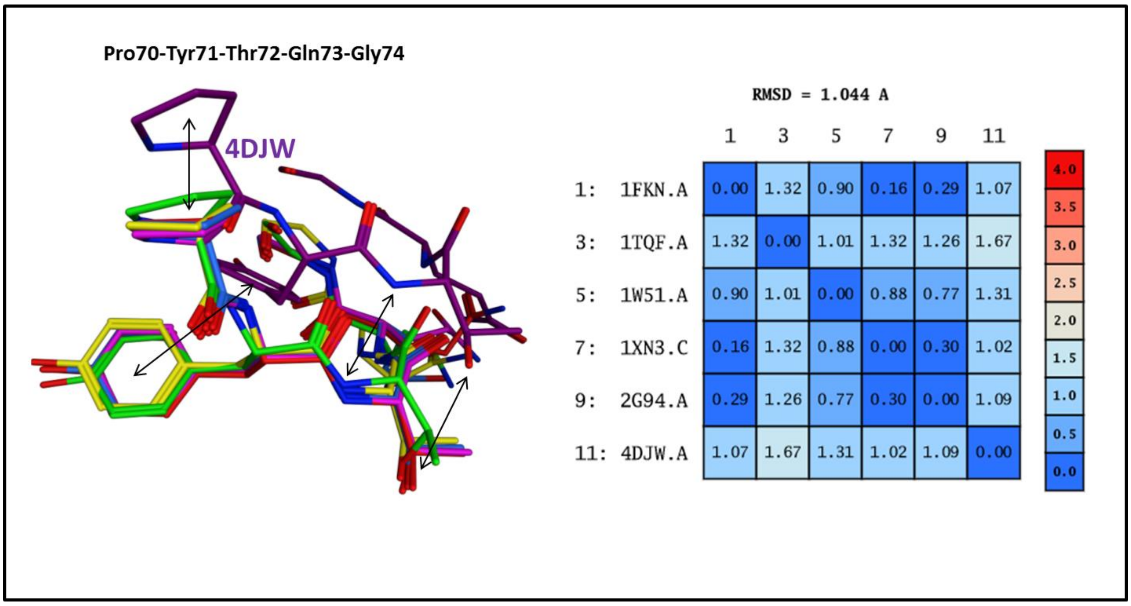

Figure S9. Six Flap residues (Pro70-Tyr71-Thr72-Gln73-Gly74) of superimposed structures (by Cα pocket residues): 2G94, 1XN3, 1TQF, 1FKN, 1W51 and 4DJW. 4DJW (in purple) differs significantly in the flap region. Colors: 1TQF (yellow), 1W51 (green), 2G94 (pink-violet), 1XN3 (blue) and 1FKN (red).

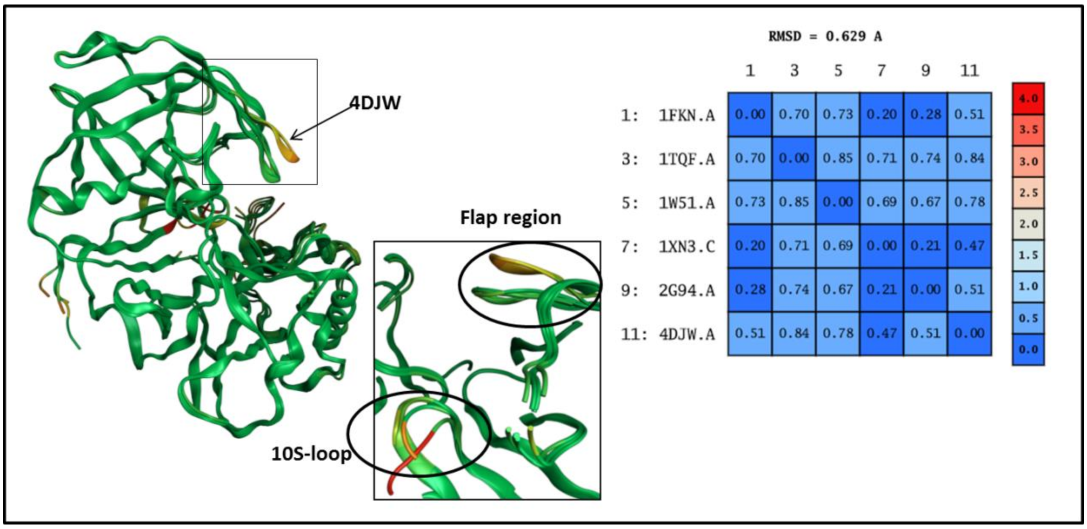

Figure S10. Six Superimposed structures (Cα, all residues): 2G94, 1XN3, 1TQF, 1FKN, 1W51 and 4DJW. Colors of chains by RMSD. 4DJW differs significantly in the flap region. Regions with different RMSD in the six structures (not including those that result from a chain break): 10S loop and the flap.

Table S5: In vitro results on BACE-1 and AChE for 36 selected molecules

Vendor	Name	Inhibition of BACE % @100 µM	Inhibition of AChE % @10 µM
**IBS**	STOCK1N-48486	7.4	-7.3
**IBS**	STOCK1N-53097	-6.2	-28
**IBS**	STOCK1N-53212	-23.1	3.0
**IBS**	STOCK1N-61184	3.6	7.1
**IBS**	STOCK1N-61193	9.0	4.9
**IBS**	STOCK1N-69309	37.3	9.0
**IBS**	STOCK1N-69840	26.9	12.3
**ChemDiv**	C066-2423	24.6	-2.7
**ChemDiv**	E155-1019	10.4	-29.2
**ChemDiv**	C561-2749	7.0	6.2
**ChemDiv**	E216-5553	23.5	-16.8
**ChemDiv**	C700-2595	19.4	**64.8**
**ChemDiv**	E216-5769	22.6	-19.8
**ChemDiv**	C741-0335	5.5	**75.6**
**ChemDiv**	F681-0222	**69.3**	**63.7**
**ChemDiv**	C798-0987	24.6	4.3
**ChemDiv**	F681-0412	**65.5**	**36.3**
**ChemDiv**	D486-0117	32.4	11.6

**ChemDiv**	F936-0709	52.2	-28.6
**ChemDiv**	E015-0847	-0.4	16.2
**ChemDiv**	T991-0560	21.3	-23.2
**ChemDiv**	E015-1455	9.9	24.7
**ChemDiv**	Z274-0419	**75.7**	-11.7
**Enamine**	T5324893	fluorescent	-16.0
**Enamine**	T6432876	2.6	3.5
**Enamine**	T5625480	30.4	-1.6
**Enamine**	T5698547	14.2	19.1
**Enamine**	T5377302	12.4	8.4
**Enamine**	T5744134	8.5	-7.1
**Enamine**	T5477385	-24.5	9.5
**Enamine**	T5744160	-3.9	-6.6
**Enamine**	T5571247	38.2	11.3
**Enamine**	T5988292	16.1	2.0
**Enamine**	T6769894	**81.9**	18.8
**Enamine**	T6009657	45.3	0.2
**Enamine**	T7116048	-21.6	6.5

Numbers in bold: high activity values

Table S6: Tanimoto similarity values matrix for hit compounds 1-6

Cmp no.	1	2	3	4	5	6
**1**	**1**	0.71	0.69	0.38	0.35	0.23
**2**	0.71	**1**	0.98	0.31	0.43	0.26
**3**	0.69	0.98	**1**	0.30	0.43	0.26
**4**	0.38	0.31	0.30	**1**	0.24	0.25
**5**	0.35	0.43	0.43	0.24	**1**	0.30
**6**	0.23	0.26	0.26	0.25	0.30	**1**

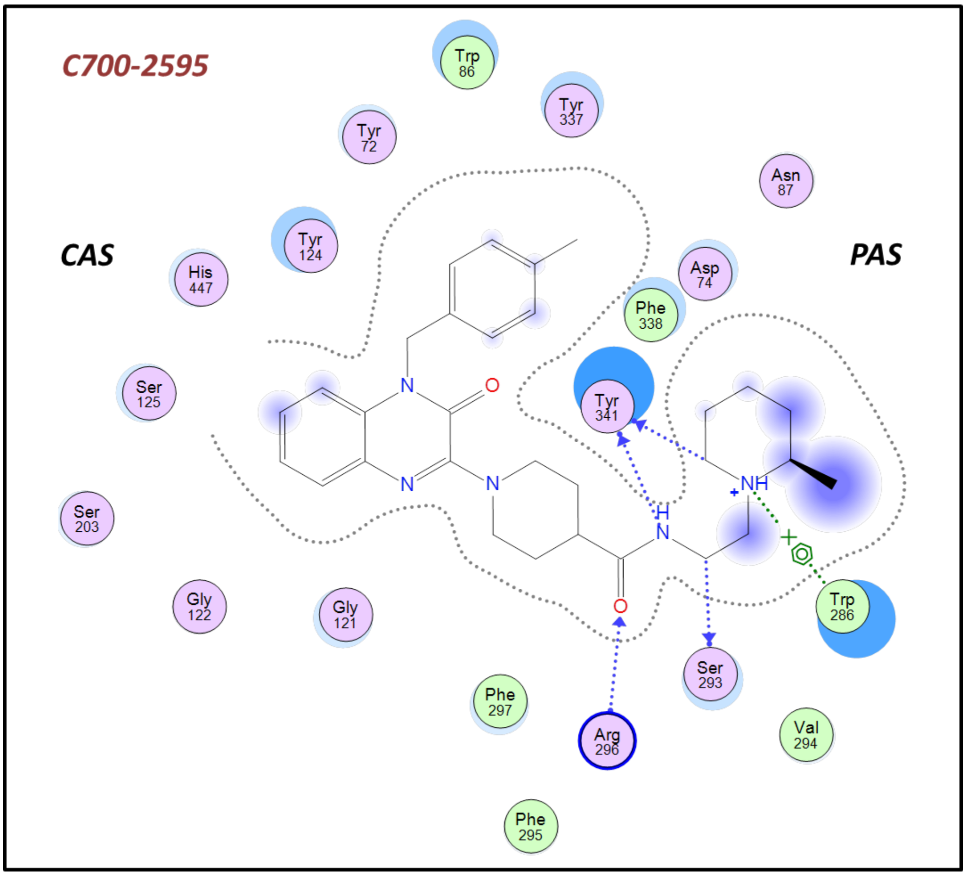

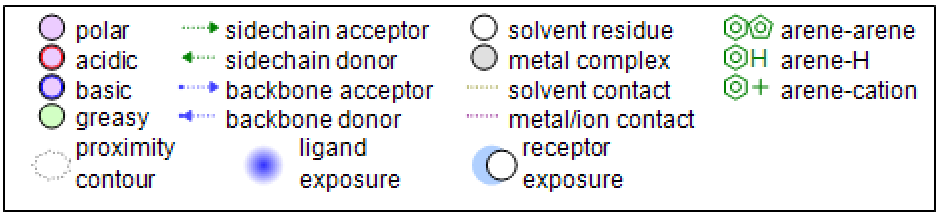

Figure S11. A docking pose of AChE hit, **1** in MOE. The quinoxalinone ring with an aromatic substituent was located in the CAS region, while the protonated nitrogen on piperidine moiety was docked in the PAS.

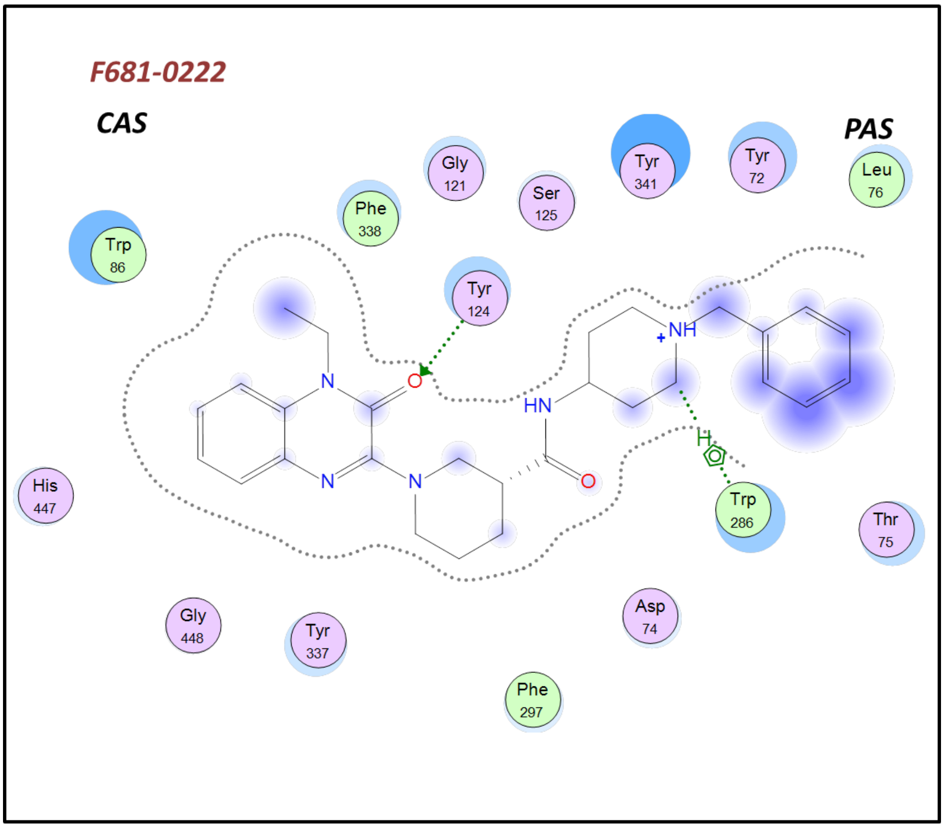

Figure S12. A docking pose of AChE hit, **2** in MOE. The quinoxalinone ring was located in the CAS region, while the protonated nitrogen on piperidine moiety was docked in the PAS.

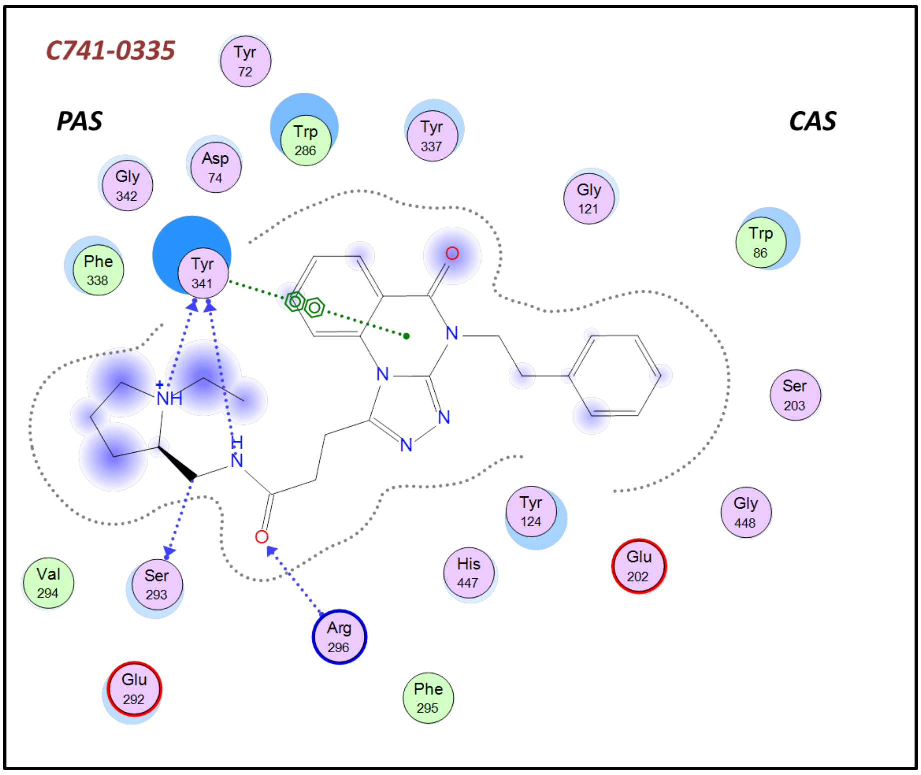

Figure S13. A docking pose of AChE hit **4** in MOE. The protonated nitrogen on pyrrolidine moiety was docked in the PAS, while an aromatic moiety in the CAS.

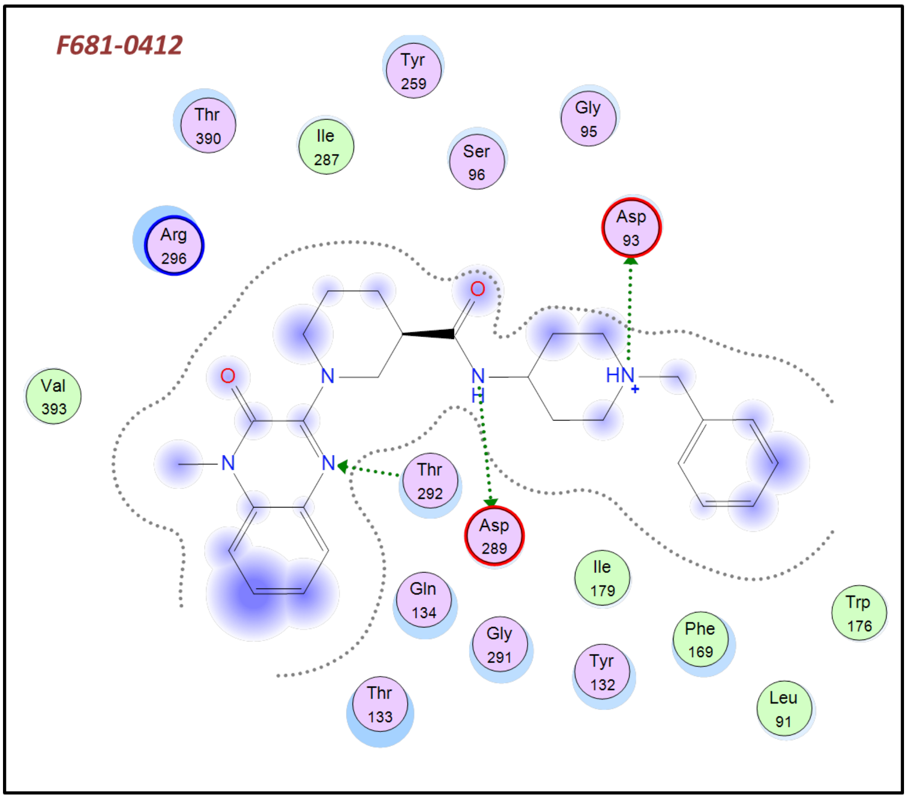

Figure S14. Docking pose of the hit molecule **3** in BACE-1 Asp93 deprotonated structure shown in MOE. Both of the catalytic Aspartates form hydrogen bonds with the hit molecule.

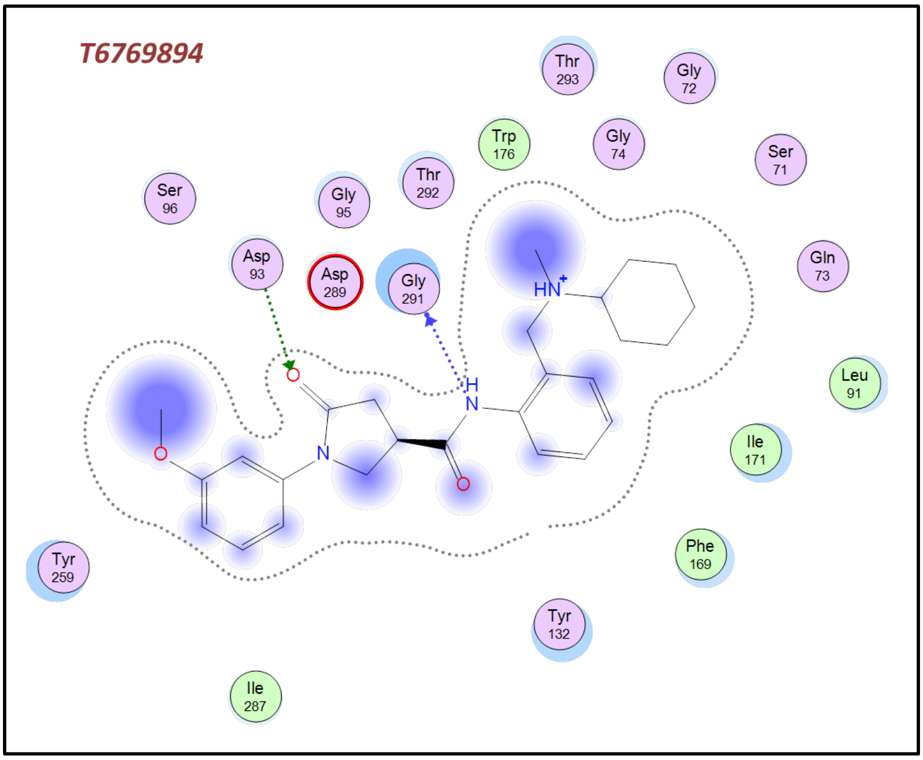

Figure S15. Docking pose of the hit molecule **5** in BACE-1 Asp93 protonated structure, shown in MOE.

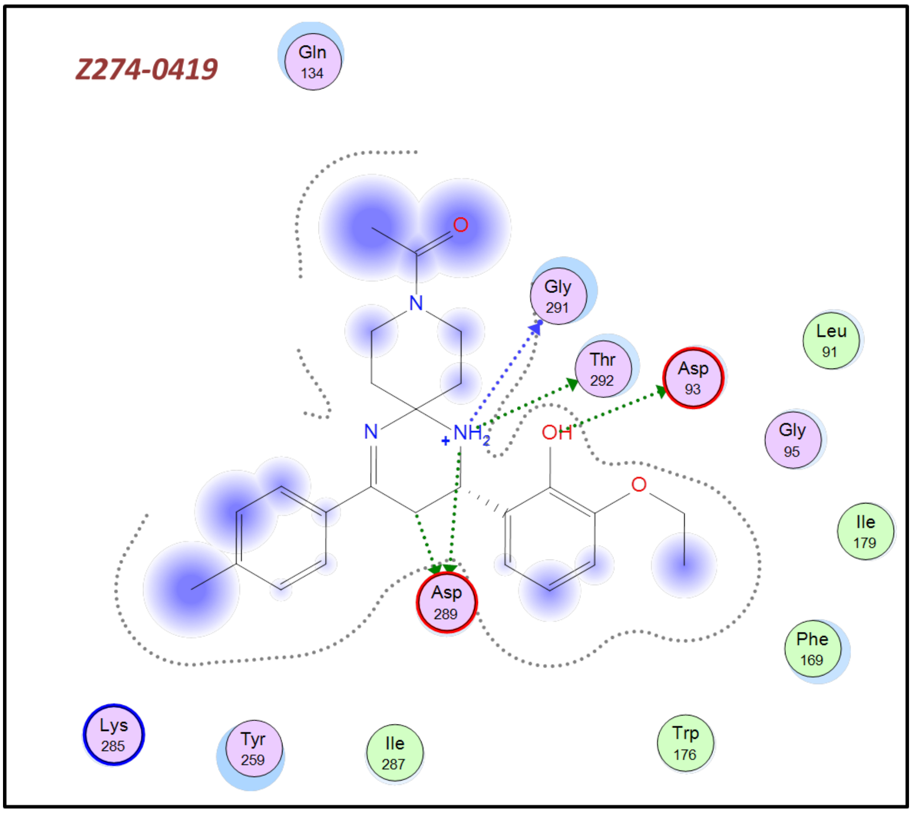

Figure S16. Docking pose of the hit molecule **6** in BACE-1 Asp93 protonated structure, shown in MOE.

A New Dual Model for AChE- and BACE-1- Active Molecules

A new ISE model for dual inhibitors was built, based on a small training set of 48 molecules active on **both** AChE and BACE-1 using a similar procedure to the models described in this manuscript. Our aim was to examine if there is a better performance for one dual model compared to combination of several models for different targets. Specifically, two sets of AChE (ChEMBL220) and for BACE-1 (ChEMBL4822) inhibitors were downloaded from CHEMBL (with published molecules till 2014). Both sets contained all the existing activity types (IC_{50}, K_i, inhibition etc.) in order to obtain the largest set possible. The initial molecular lists contained 7700 BACE-1 ligands and 9242 AChE ligands. These lists were united and only the name duplicates of AChE-BACE-1 pairs were kept (139 pairs). Then, molecules with the comments "not active", "not determined" and "outside typical range" were removed and 84 molecule pairs remained. The obtained list was filtered according to activity values with the following cutoffs: IC_{50} = 50 μM, K_i = 16,666 nM and inhibition = 60%. Since only 32 pairs of molecules remained, 20 other molecular pairs with dual activity were added from papers.[20-26] After similarity-filtering by Tanimoto=1 the final list included 48 molecules with dual activity (see pdf and excel files for the active dual molecules). A list of 11,580 random molecules from ZINC was added as inactives (after applicability domain filtering according to the following four MOE descriptors: lip_acc 3-9, lip_don 1-6, logP 3.18-9.82, weight 315-765 gr/mol). An ISE- actives vs. randoms model was built in a similar way to the models described here.

The screening of external databases (Enamine, ChemDiv etc.) included filtration by the dual model, then by the MOE FCharge descriptor with value = +2. This was due to expected better aqueous solubility and due to the requirement of binding to negative Asp in BACE-1 and aromatic residues in AChE). Finally, FlexX docking was performed. The molecules with the highest index for the dual model, 0.943, which were 928 molecules out of 17,633, were chosen. Only 76 molecules that had a charge of +2 were docked into AChE and BACE-1 structures. 16 molecules were docked (based on the existence of important interactions) by FlexX in both structures and therefore chosen for biological tests, and four other molecules weren't docked in AChE, but were added to the final list of molecules, due to relatively good results in BACE-1 docking (i.e., docked in three BACE-1 structures, or having 17-20 docked poses out of 20 for two of the structures, each with maximum 10 poses). Eventually, 19 docked molecules

were chosen for enzymes tests and seven of them, Enamine molecules, were tested in AChE and BACE-1 assays (Figure S16). None of the molecules was active on AChE or BACE-1, though it was sometimes impossible to know due to fluorescence of the molecules or precipitation (Table S7).

To find an explanation for negative results, these selected molecules were filtered with the previous separate model filters of AChE and BACE-1 to examine if there are differences in the indexes comparing to the new dual model. Indeed, there were higher indexes for actives vs. randoms models, but low negative indexes for high vs. low models for both AChE and BACE-1 (Table S8). Additionally, these inactive molecules were (except one) docked in AChE and BACE-1 crystal structures. Therefore, it is concluded that screening with separate AChE and BACE-1 models, each based on many inhibitors is better than screening with one model based on a small set of dual inhibitors. Additionally, docking procedure by itself is not sufficient for finding new hits, but the combination of docking and screening with several models, including higher high vs. low model indexes has a better chance to lead to the requested results.

Table S7. In vitro BACE-1 results for seven dual AChE-BACE-1 model screening candidates from Enamine

Molecule Name	BACE-1 %Inhibition@100 μM	Comment
**T0501-5561**	173	Fluorescent
**T5646299**	-41	Activating?
**T5434683**	51	Partially precipitating
**T5374169**	12	/
**T0507-1256**	134	Fluorescent
**T0505-4322**	236	Fluorescent
**T0506-2176**	42	/
**BACE-1 inhibitor IV.**	100	Control at 1 μM

Table S8: Models Indexes and docking results for seven Enamine candidates from dual model screening

Molecule Name	Dual Index[a]	AChE Indexes			BACE-1 Indexes		AChE/ BACE Poses[c]
		Model 1[b]	Model 4	Model 5	Model 2	Model 8	
**T0501-5561**	**0.94**	0.88	-0.58	-0.71	0.21	-0.58	1/11
**T0505-4322**	**0.94**	0.88	-0.67	-0.76	0.21	-0.74	6/2
**T0506-2176**	**0.94**	0.88	-0.51	-0.67	0.41	-0.71	0/15
**T0507-1256**	**0.94**	0.87	-0.12	-0.28	0.83	-0.70	2/1
**T5374169**	**0.94**	0.88	-0.61	-0.73	-0.67	-0.78	3/5
**T5434683**	**0.94**	0.88	-0.59	-0.74	0.21	-0.59	7/2
**T5646299**	**0.94**	0.73	-0.72	-0.74	0.18	-0.58	1/1

[a] Index of the dual AChE-BACE-1 model. The candidate molecules were screened by this model.; [b] index of AChE model no. 1. The candidate molecules weren't screened by this or by any "separate" model for AChE or BACE-1, but only passed through each separate model's filters to check the index in comparison to the dual model; [c] The sum of poses for three BACE-1 structures, while for AChE there was one structure. Therefore, the maximum number of poses for AChE is 10 and for BACE-1 is 30. Number of poses in AChE are on the left, for BACE-1 - on the right.

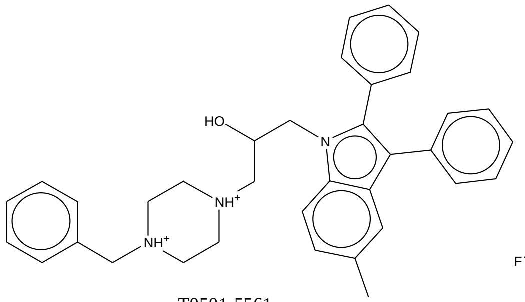

T0501-5561

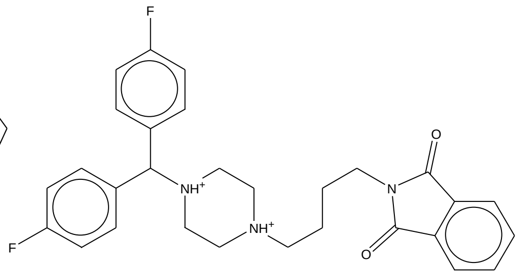

T5646299

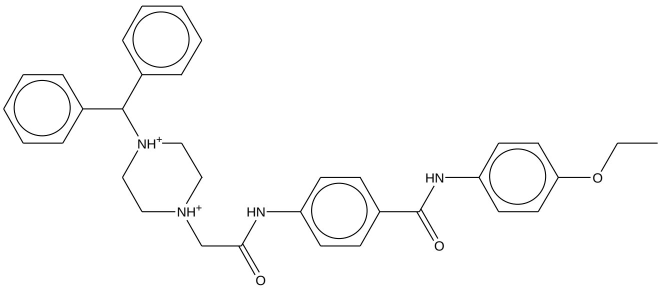

T5434683

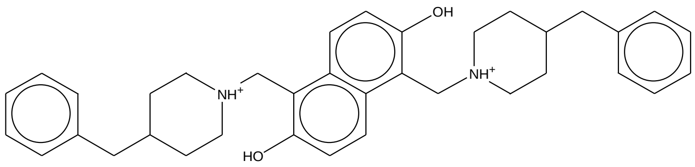

T0507-1256

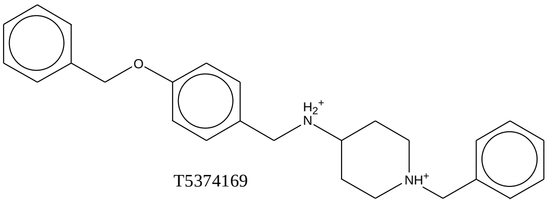

T5374169

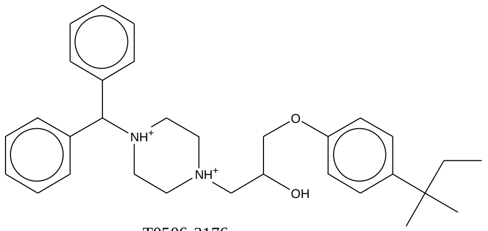

T0506-2176

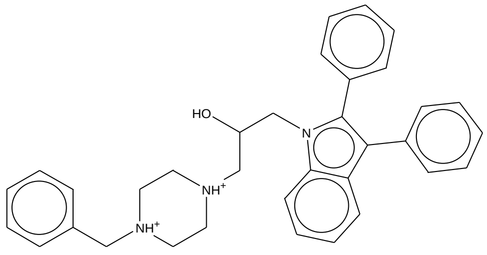

T0505-4322

Figure S17. Seven Enamine molecules screened by the dual model and docking. All found inactive on AChE and BACE-1.
